# Cell generation dynamics underlying naive T-cell homeostasis in adult humans

**DOI:** 10.1371/journal.pbio.3000383

**Published:** 2019-10-29

**Authors:** Jeff E. Mold, Pedro Réu, Axel Olin, Samuel Bernard, Jakob Michaëlsson, Sanket Rane, Andrew Yates, Azadeh Khosravi, Mehran Salehpour, Göran Possnert, Petter Brodin, Jonas Frisén

**Affiliations:** 1 Department of Cell and Molecular Biology, Karolinska Institute, Stockholm, Sweden; 2 Center for Neuroscience and Cell Biology, University of Coimbra, Coimbra, Portugal; 3 Science for Life Laboratory, Department of Women’s and Children’s Health, Karolinska Institutet, Solna, Sweden; 4 Institut Camille Jordan, CNRS UMR 5208, University of Lyon, Villeurbanne, France; 5 Center for Infectious Medicine, Department of Medicine, Karolinska Institutet, Karolinska University Hospital Huddinge, Stockholm, Sweden; 6 Department of Pathology and Cell Biology, Columbia University Medical Center, New York, New York, United States of America; 7 Institute of Infection, Immunity & Inflammation, Glasgow Biomedical Research Centre, University of Glasgow, Glasgow, United Kingdom; 8 Department of Physics and Astronomy, Ion Physics, Uppsala University, Uppsala, Sweden; 9 Department of Newborn Medicine, Karolinska University Hospital, Stockholm, Sweden; National Cancer Institute, UNITED STATES

## Abstract

Thymic involution and proliferation of naive T cells both contribute to shaping the naive T-cell repertoire as humans age, but a clear understanding of the roles of each throughout a human life span has been difficult to determine. By measuring nuclear bomb test–derived ^14^C in genomic DNA, we determined the turnover rates of CD4^+^ and CD8^+^ naive T-cell populations and defined their dynamics in healthy individuals ranging from 20 to 65 years of age. We demonstrate that naive T-cell generation decreases with age because of a combination of declining peripheral division and thymic production during adulthood. Concomitant decline in T-cell loss compensates for decreased generation rates. We investigated putative mechanisms underlying age-related changes in homeostatic regulation of CD4+ naive T-cell turnover, using mass cytometry to profile candidate signaling pathways involved in T-cell activation and proliferation relative to CD31 expression, a marker of thymic proximity for the CD4+ naive T-cell population. We show that basal nuclear factor κB (NF-κB) phosphorylation positively correlated with CD31 expression and thus is decreased in peripherally expanded naive T-cell clones. Functionally, we found that NF-κB signaling was essential for naive T-cell proliferation to the homeostatic growth factor interleukin (IL)-7, and reduced NF-κB phosphorylation in CD4^+^CD31^−^ naive T cells is linked to reduced homeostatic proliferation potential. Our results reveal an age-related decline in naive T-cell turnover as a putative regulator of naive T-cell diversity and identify a molecular pathway that restricts proliferation of peripherally expanded naive T-cell clones that accumulate with age.

## Abstract 2

Our pool of naive T cells is critical for protection against new infections and cancers. By measuring remnant 14C from 1960s nuclear bomb blasts that has been incorporated into cellular DNA, this study defines the average age of the naive T-cell pool in healthy adults, revealing the slow, regulated turnover of the naive T-cell pool, supporting its maintenance for a human lifetime.

## Introduction

Naive T-cell numbers and clonal diversity represent the adaptive immune system’s potential to sense and respond to foreign pathogens and mutant proteins expressed by malignant cells [1, 2]. In humans, the naive T-cell pool is established primarily in the first decade of life through the massive efflux of billions of newly produced naive T cells from the thymus [3–5]. Each new naive T cell is uniquely defined by a clonally heritable T-cell receptor, which confers specificity for a restricted set of peptides for which a T cell is capable of sensing and responding against [6]. Thus, the number of unique foreign peptides that the T-cell pool can sense is proportional to the number of unique clones present in the naive T-cell population at any given time.

It has long been recognized that the thymus undergoes a dramatic involution between birth and early adulthood in humans [4, 7]. Loss of thymic output in adults shifts the responsibility for the maintenance of naive T-cell numbers to peripheral division of existing clones rather than de novo production of new, unique clones [8]. In theory, this should lead to a gradual loss of naive T-cell diversity, as individual clones compete for space and limited homeostatic growth factors, such as interleukin (IL)-7, in the peripheral lymphoid tissues [9, 10]. Surprisingly, diversity and naive T-cell numbers are largely maintained until roughly 65 years of age in most humans, with only CD8^+^ naive T cells showing an age-related decline in numbers during this period [10–13]. Notably, a dramatic loss of diversity has been observed in a fraction of elderly individuals, which has led to speculation that a sudden collapse of naive T-cell diversity may be causally linked to immune dysfunction associated with advanced age [10, 11].

Naive T cells appear to divide rarely relative to other hematopoietic lineages, offering a putative mechanism for the maintenance of diversity over long periods of time [14]. Because naive T cells divide infrequently, defining their actual turnover rates has been challenging, with estimates ranging from months to decades in different studies based on short-term pulse-chase labeling studies [15]. A major confounding feature of these studies is that only a very small fraction of the dividing naive T-cell population is labeled in the uptake period, and often no dilution of the label is observed in the short chase periods during which each human volunteer can be monitored [14, 16]. Additionally, recent evidence points toward greater heterogeneity within the classically defined “naïve" T-cell compartment, with some cell types present in this population exhibiting higher turnover than others, potentially leading to more rapidly dividing non-naive cells being labeled and measured in these studies [1, 17]. Heterogeneity within truly naive T-cell populations is also known to exist. The markers CD31 and CD103 can subdivide CD4^+^ and CD8^+^ naive T cells, respectively, into fractions with distinct replication histories defined by variable content of T-cell receptor excision circles (TRECs), which are formed during naive T-cell development and diluted upon peripheral divisions [18, 19]. However, interpretation of these findings is complicated by the fact that differing TREC content may reflect thymic production of new T cells or selective outgrowth of certain clones in the periphery [20–22]. This also underscores the importance of combining TREC content with accurate measurements of cell turnover rate when determining thymic activity in adult humans [23–25].

**Fig 1:**
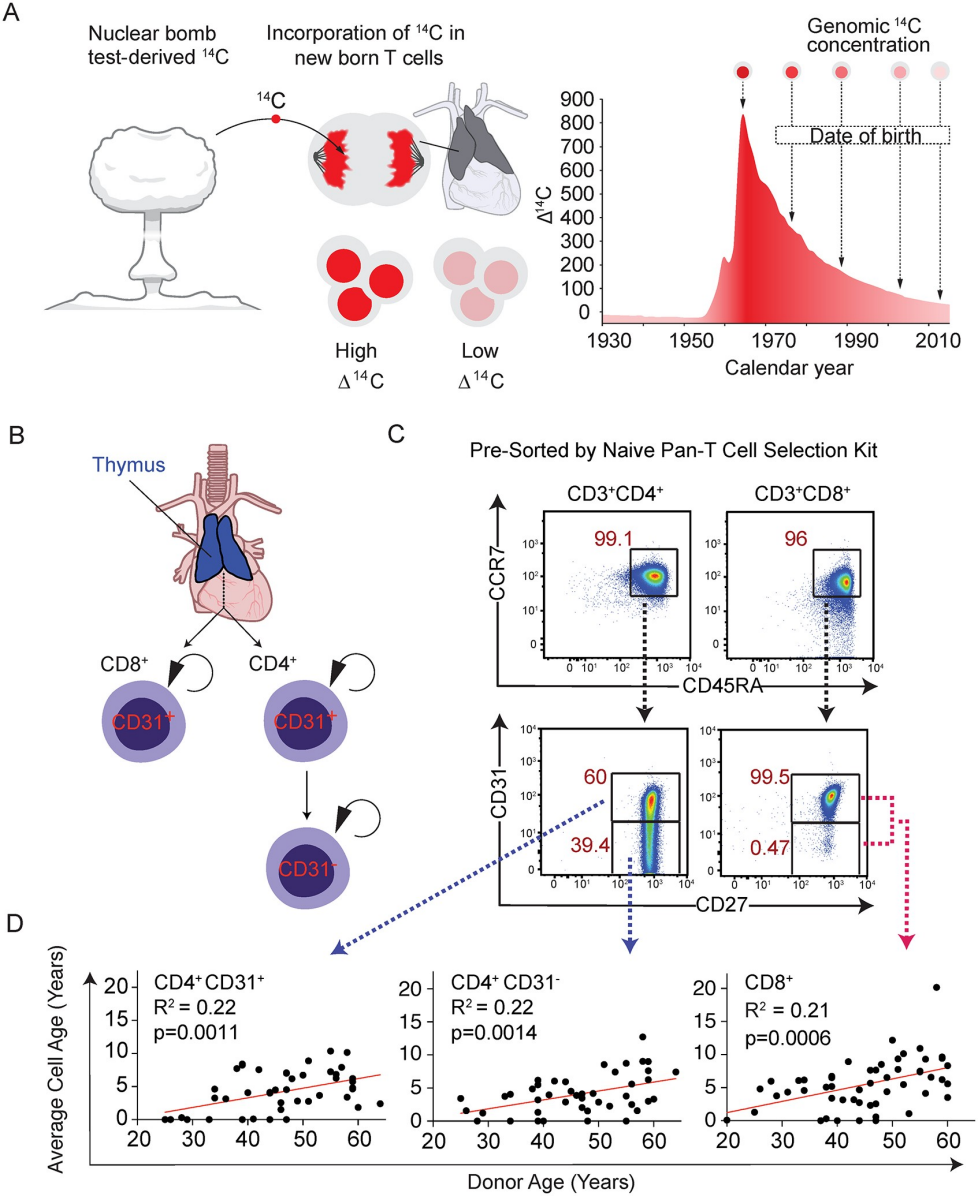
^14^C measurements of naive T-cell populations reveal age-dependent decrease in naive T-cell turnover. (A) Schematic explaining how nuclear bomb test–derived ^14^C is incorporated into the DNA of newly produced naive T cells (either in the thymus or through cell division in the periphery). Depending on what year a new naive T cell is formed, the ^14^C content will mirror atmospheric levels, providing a cellular birth date. (B) Populations of naive T cells surveyed in this study include total CD8^+^ T cells and both CD4^+^CD31^+^ and CD31^−^ T-cell populations. CD8^+^ and CD4^+^CD31^+^ naive T cells are presumed to originate in the thymus and divide in the periphery with CD4^+^CD31^+^ cells, yielding CD4^+^CD31^−^ cells in the peripheral tissues. (C) Flow cytometry plots depicting gating strategies for purifying each population of naive T cells. Total CD3^+^ cells are shown for each gate, which were obtained after purifying all naive T cells from buffy coats subjected to red blood cell lysis. CD8^+^ naive T cells are >98% CD31^+^, so no distinction was made for sorting this population. (D) Average cell DNA age determined by ^14^C content analysis depicted for each population relative to donor age. Increased cell DNA age is observed for all populations relative to donor age (Student *t* test). CCR7, C-C chemokine receptor 7.

To better define the turnover rates of naive T cells in healthy adult humans, we characterized the average cellular DNA age of millions of sort-purified naive T cells isolated from 59 healthy adults between 20 and 64 years of age by retrospective ^14^C dating of DNA [26]. Retrospective birth dating takes advantage of the large increase in atmospheric ^14^C due to nuclear bomb tests during the Cold War [27]. The ^14^C generated by the nuclear detonations reacted with oxygen to form ^14^CO_2_, which is taken up by plants through photosynthesis. The atmospheric ^14^C levels are mirrored in the human body at any given time, as we eat plants or animals that consume plants. Dividing cells incorporate ^14^C with a concentration corresponding to atmospheric ^14^C levels, creating a stable date stamp in the genomic DNA, which can be used to assess the time between cell divisions as defined by new DNA synthesis and calculate turnover dynamics (Fig 1A). In this way, changes in ^14^C levels in the environment, and consequently the DNA of newly produced cells, function as a lifelong pulse-chase experiment impacting all cells of an organism. This strategy has been successfully used to measure the rates of turnover of long-lived cells in humans including neurons, oligodendrocytes, adipocytes, cardiomyocytes, and plasma cells [26, 28–31].

Combining our cell DNA age determinations with reported changes in peripheral naive T-cell number and TREC content, we provide a complete overview of how thymic activity, homeostatic division rates, and naive T-cell loss rates shape the peripheral naive T-cell pool throughout adulthood. We show for the first time, to our knowledge, that the entire naive T-cell pool undergoes constant turnover in the periphery in healthy adult humans. We observed that turnover rates for both CD4^+^ and CD8^+^ naive T cells decreased relative to donor age, consistent with recent predictions from mouse models [32]. Mathematical modeling shows that this decreased turnover was due to gradually reduced cell loss and peripheral division rates of naive T cells with aging. For CD4^+^ naive T cells, we determined the average cell DNA age for purified CD4^+^CD31^+^ recent thymic emigrants and CD4^+^CD31^−^ peripherally expanded naive T-cell populations. The use of CD103 to subdivide CD8^+^ naive T cells, which is typically expressed by less than 1% of circulating naive T cells, was not possible, because of restrictions on the numbers of cells required for accurate ^14^C testing. Modeling results indicated that CD31^−^ naive T cells may be limited in their ability to undergo additional homeostatic proliferation, whereas CD31^+^ naive T cells undergo peripheral homeostatic proliferation. This hypothesis was confirmed both in vivo and in vitro, and we provide a molecular mechanism that is based on differences in nuclear factor κB (NF-κB) phosphorylation relative to CD31 expression and can account for this observation. Such a mechanism may in part explain how clonal diversity is preserved despite peripheral clonal competition for resources throughout decades of adult life. Our findings provide the first direct measurements of naive T-cell life spans throughout adulthood in humans and identify a potential mechanism that may in part explain the slow, regulated turnover of this population in the peripheral tissues.

## Results

### Total CD4^+^ and CD8^+^ naive T-cell turnover rates decrease relative to donor age

In order to obtain accurate ^14^C measurements, we required at least 3.5 million purified naive T cells. CD4^+^ naive T cells are typically more numerous in the blood, and the fraction of CD31^+^ and CD31^−^ cells within this population typically afforded us the ability to obtain sufficient cell numbers for each. CD8^+^ naive T cells were isolated as a single population because of cell number constraints (Fig 1B and 1C). A two-step purification strategy was developed to circumvent density centrifugation steps, required to remove granulocytes and red blood cells, which introduced contamination to downstream ^14^C measurements. After red blood cell lysis, total white blood cell samples were subjected to a pan-naive T-cell isolation by magnetic beads (which also removes CD25^+^ naive regulatory T cells) followed by fluorescence-activated cell sorting (FACS) purification of CD45RA^+^CCR7^+^ CD4^+^CD31^+^, CD31^−^, or CD8^+^ T cells (Fig 1C, S1 Table). For CD8^+^ naive T cells, we included a subset of donors, which were collected early in our analysis using CD45RA^+^CD62L^+^CD3^+^CD8^+^ as a purification criteria after we concluded that the inclusion of these samples did not impact our downstream results (S1 and S2 Tables). Notably, these definitions of naive T cells include a small fraction of memory T cells, known as “stem cell memory” T (Tscm) cells, which divide roughly once every 2–4 years and typically represent less than 5% of the naive T-cell pool [17, 33, 34]. Because of their relative scarcity compared to true naive T cells, as well as their comparable turnover rates, they are unlikely to have a meaningful impact on ^14^C measurements, which represent averages of the total population.

We measured the ^14^C concentration in DNA isolated from purified naive T cells from each population for all donors when possible, by accelerator mass spectrometry (S1F Fig, S1 Table). The ^14^C content of an individual cell reflects both the ^14^C content of the 50% of its DNA that is inherited directly from its parent and the ^14^C content of newly synthesized DNA, which is assumed to reflect current atmospheric levels [26]. Despite the complexity of this process, it can be shown that the ^14^C content of a pool of cells at steady state reflects the time since a cell was produced on average, whether it be from the thymus or through homeostatic division.

To illustrate this, we consider a T cell in the periphery with an average DNA age of x years. If the T cell divides, the two daughter cells will inherit an average DNA age equal to x/2 on average. If a cell is produced in the thymus, with a DNA age of 0 years, the resulting average will be (x + 0)/2 = x/2 also (a detailed supplemental guide to all mathematical modeling is included entitled “Supplemental mathematical models” [S1 Text]) [26]. In simple terms, for a population at steady state, the average cell DNA age is the inverse of the rate of turnover and is also the mean life span of all cells in the population.

meanlifespan=averagecellDNAage=1/turnoverrate (1)

At steady state, the balance between the loss and production of new cells from the thymus or by peripheral division preserves the distribution of cell DNA age within the population. If the timescale over which a population undergoes turnover is short compared to the individual’s life span, we can use this cell DNA age distribution together with the time course of recent levels of atmospheric carbon to estimate the mean of this distribution and hence the average life span of naive T cells in an individual.

Stratifying these estimates by donor age, we observed that the mean DNA age of CD4^+^CD31^+^, CD4^+^CD31^−^, and CD8^+^ naive T cells all increased relative to donor age (Fig 1D, linear regression versus donor age: all *p* < 0.003). For all naive T cells, the mean intervals between cell divisions were of the order of 0–6 years (mean: 1.7 years, SD: 2.1 years) in 20–30-year-olds and 0–19 years (mean: 6.6 years, SD: 3.4 years) in 50–65-year-olds. This corresponds to an average annual turnover rate of 59% in 20–30-year-olds and 15% in 50–65-year-old donors (*t* test, *p* < 1 × 10^−6^) (Fig 1D, S2 Table). Robustness analysis (S3 Table) confirms this analysis; when using datasets perturbed by noise, correlation was maintained even at high noise levels, and donor-age random permutations removed the correlation. We observed no statistically significant differences in naive T-cell DNA age with respect to donor’s gender.

### The entire naive T-cell pool undergoes constant turnover throughout life

The heterogeneous nature of the naive T-cell population [1] complicates the quantification of its homeostatic dynamics. In addition to phenotypically distinct cell types occupying the classical naive T-cell population, individual clones may exhibit differences in longevity or their propensity to respond to homeostatic growth signals [35, 36]. Dilution of TRECs across multiple decades of adult life could easily result from uneven clonal expansions, in which only a fraction of the naive T-cell pool is selectively expanding in the periphery [20]. Likewise, short-term labeling studies only label a small percent of the total naive T-cell population (0%–5%), meaning that no inference can be made concerning the potential of the remaining cells to undergo division [14, 16].

By measuring the average cell DNA age of millions of naive T cells from donors born at various times before, during, and after the atmospheric ^14^C bomb-spike, we can address whether the naive T-cell compartment contains subpopulations of long-lived nondividing cells. If a large fraction of naive T cells were to be produced during the first decades of life and would recirculate without undergoing subsequent homeostatic division, these cells would contain ^14^C levels in their DNA that match the environment from an individual’s childhood [26]. This would result in 14C levels from donors born during the peak of the bomb-spike to mimic atmospheric 14C levels as a result of accumulated nondividing naive T cells generated during this timeframe.

**Fig 2:**
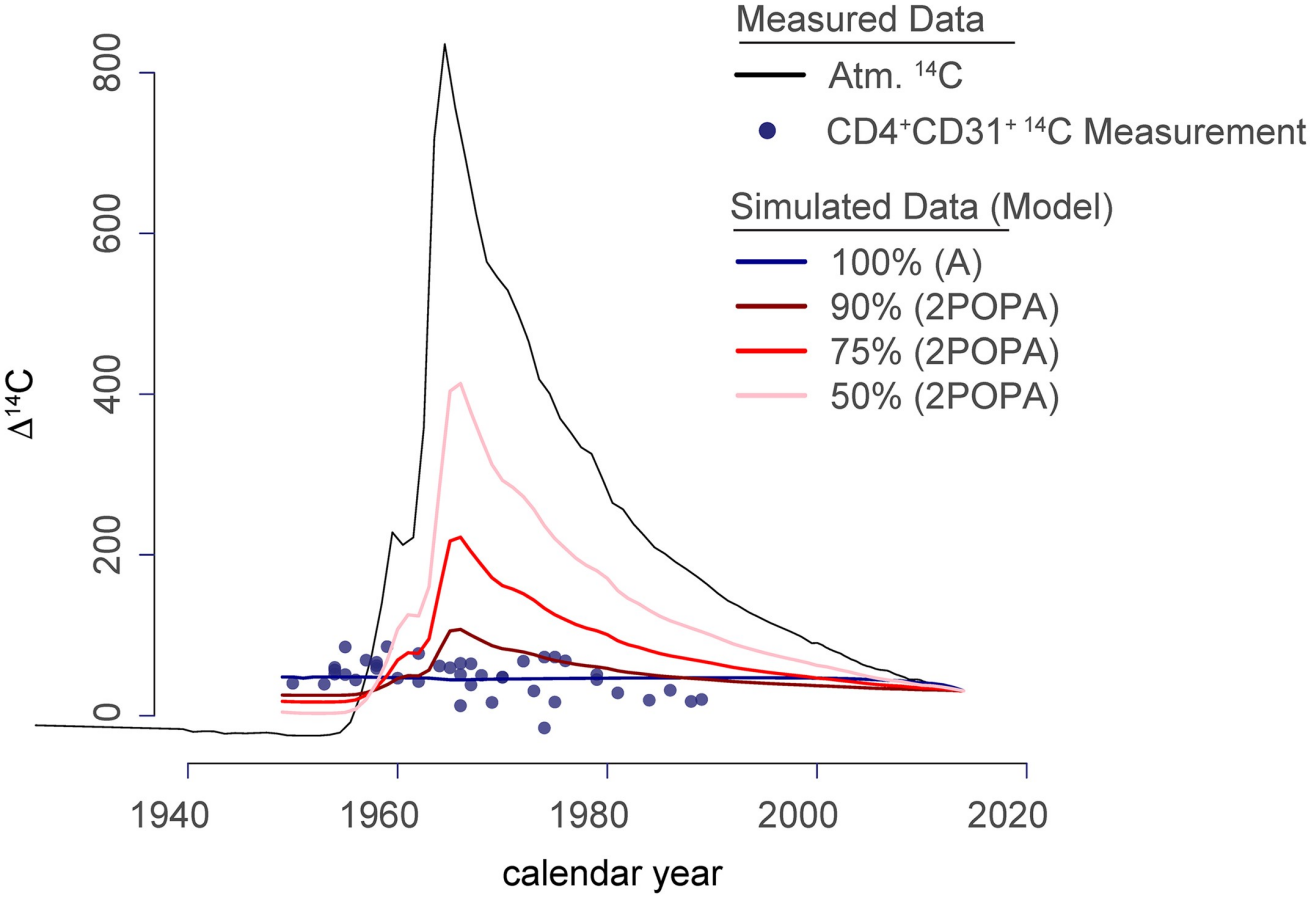
All naive T cells undergo turnover throughout adult life. Individual data points for ^14^C measurements of DNA isolated from CD4^+^CD31^+^ naive T cells plotted according to donor birth year (x-axis). The atmospheric (“Atm.”) ^14^C levels are plotted (black line), and simulated data are shown to represent the average distributions of data points if 50%, 75%, 90%, or 100% of the naive T cells undergo turnover after production. If a fraction of the naive T-cell population persisted as nondividing cells in the periphery after thymic egress, donors born between 1960 and 1980 would have elevated ^14^C content measured in the DNA of the naive T-cell population. Model testing was performed for CD4^+^CD31^+^, CD4^+^CD31^−^, total CD4^+^ naive, and total CD8^+^ naive T-cell populations, and all results were consistent with 100% turnover (S4 Table, S1 Text section “ASE”).

We compared one model in which all cells are equally likely to divide (model A) with a second model (2POPA) in which populations of different sizes accumulate in the periphery without undergoing additional rounds of division (Fig 2, S1 Text p. 12, and S4 Table). Although the ^14^C samples show a positive, linear correlation with donor age (Fig 1D), they do not follow the atmospheric ^14^C levels. The best fit for our data strongly speaks against the presence of a long-lived, nondividing population within the naive T-cell populations. (Fig 2, S1 Text section 1). Robustness analysis (S3 Table) confirms this result; when using datasets perturbed by noise, we found that model A was selected >75% of the time, even with maximal perturbation (5× the SD of individual data points). Our data therefore suggest that the majority (99%) of naive T cells are maintained dynamically throughout life through the combination of the production of new cells by the thymus, proliferative renewal of existing cells, and loss through death or differentiation.

### Modeling thymic output, peripheral division, and cell loss rates based on cell DNA age estimates

Our estimates of cell DNA age allow us the opportunity to define the dynamic turnover rates for naive T cells throughout an adult human lifetime. To do this, we incorporated regressed data taken from publications for both CD4^+^ and CD8^+^ naive T cells relating to cell numbers [13], TRECs [8, 23], and cell DNA age estimates defined by ^14^C measurements (S2 Fig, S1 Table). Individually, each of these measurements gives insight into naive T-cell turnover throughout life. Healthy humans ranging from 20 to 65 years of age show minimal decline in CD4^+^ naive T-cell numbers, whereas CD8^+^ naive T-cell numbers progressively decline in this timeframe [13]. TREC content measurements in sorted naive T cells allow insights into cell loss and proliferation [24]. Finally, total cell DNA age measurements allow us to demonstrate that the entire naive T-cell pool is subject to division and define the average rate of division for any naive T cell. Using a series of linear equations, similar to those previously used to define T-cell dynamics during the first decades of human life [5, 37], we set out to quantify the processes that impact the maintenance of naive T-cell numbers and diversity throughout adult life. For cell numbers and TREC content, we based our calculations on regressed data from published studies documenting age-dependent changes in naive T-cell subsets in normal healthy adults.

**Fig 3:**
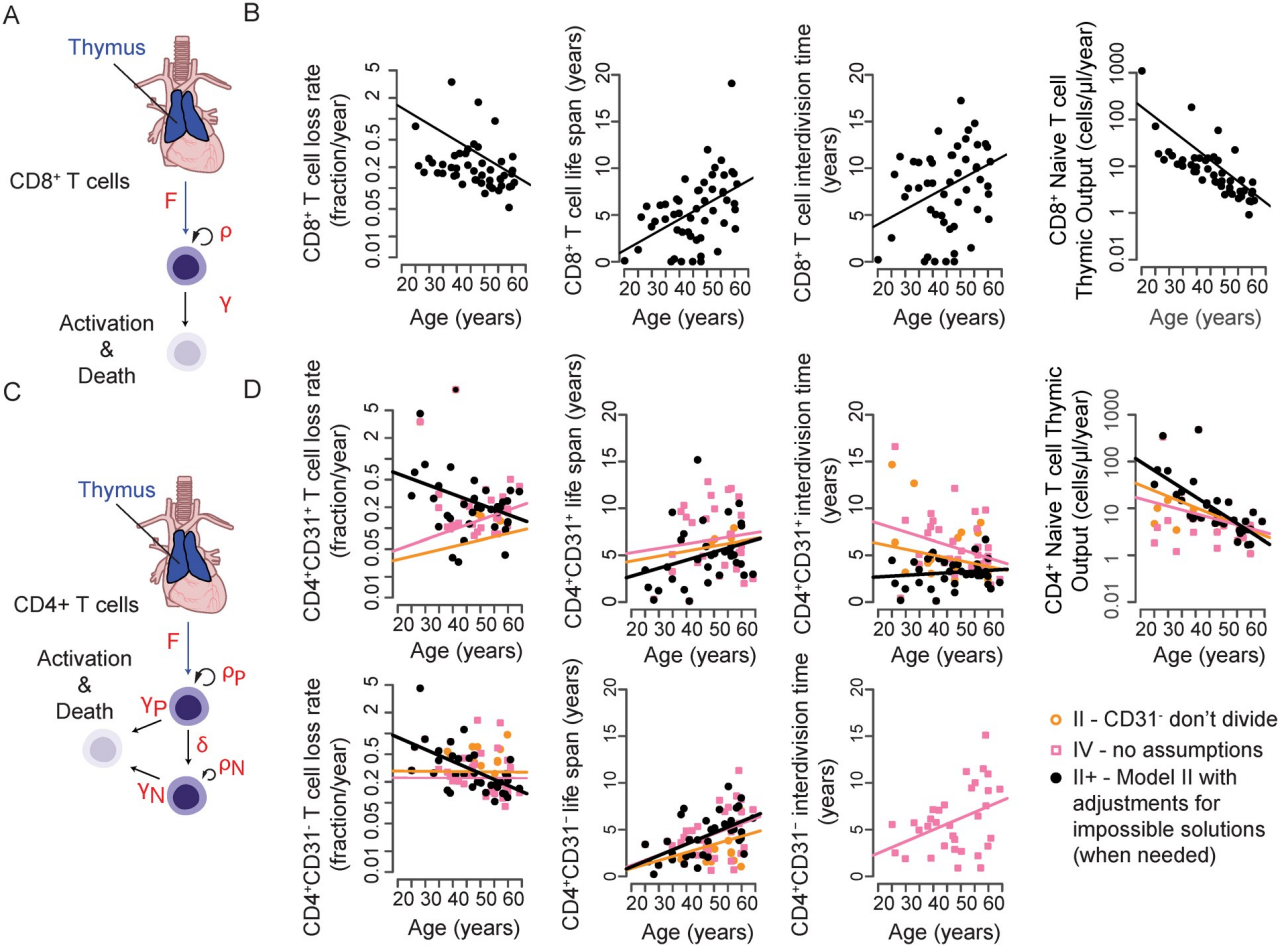
Dynamics of naive T-cell homeostasis determined by linear models. (A) Individual variables measured with linear equations depicted for CD4^+^ naive T-cell populations. (B) Solutions for linear models are shown for selected scenarios tested (from Table S3). Scenario IV (pink squares) shows solutions when no predictions are made and each dynamic variable is solved with no additional assumptions. Scenarios II and II+ show solutions when CD31^−^ naive T cells are assumed to be a nondividing population. Scenario II+ is modified to adjust for individuals when an exact solution could not be made (*n* = 7/41 donors, S1 Text, S2 Table). (C) Individual variables measured with linear equations depicted for CD8^+^ naive T-cell populations. (D) Solutions for each dynamic variable with no additional assumptions made are depicted for CD8^+^ naive T cells.

For naive CD8^+^ T cells, there were four observables for each donor: (1) cell numbers estimated from Fagnoni and colleagues [13], (2) TREC content estimated from Douek and colleagues [23], (3) cell DNA age, and (4) CD31 expression, which does not undergo age-related down-regulation and thus was set to 98%, which was the average frequency in our dataset (S1 Table). We therefore had four kinetic measurements to solve for: thymic export rate (F), peripheral proliferation rate (rho), loss rate (gamma), and a small CD31 marker loss rate (described in S1 Text). It was assumed that the CD31^+^ and CD31^−^ subsets had the same age, and that the CD31^−^ T cells did not proliferate significantly (Fig 3A). Under these conditions, a unique kinetic parameter set could be found for each donor (Fig 3B, S2 Table).

CD4^+^ naive T cells had six observables and six dynamic variables to determine. Here, we assumed that CD4^+^ naive T-cell numbers remained constant between 20 and 65 years of age as is generally accepted [12, 13], estimated TREC content from den Braber and colleagues [8], and used measured cell DNA age. For CD4^+^ naive T cells, we defined individual-specific values for both CD4^+^CD31^+^ and CD4^+^CD31^−^ fractions as well as an additional dynamic variable to account for the differentiation of CD4^+^CD31^+^ cells to CD4^+^CD31^−^ (Fig 3C, S1 Table). Importantly, the processes governing loss of CD31 expression by naive CD4^+^ T cells in humans remain unknown, although it is generally assumed that the CD31^−^ population accumulates because of expansion of CD31^+^ cells coupled with down-regulation of CD31 expression with age [38]. We estimated the following variables for CD4^+^ naive T cells: the rate of export of CD4^+^CD31^+^ naive T cells from the thymus (F) and the division rates of CD4^+^CD31^+^ and CD4+CD31^−^ T cells (rho_P and rho_N, respectively). Upon division, TREC content is split with equal probability between daughter cells. There is also a probability delta for CD31^+^ T cells to lose the CD31 marker and to produce two CD31^−^ T cells. The two populations are lost though death or differentiation at rates gamma_P and gamma_N. The resulting model is expressed as an algebraic linear system of six equations and six kinetic parameters to solve for (S1 Text, Section 2 Linear Models). For each individual, conditions for existence and uniqueness of positive parameters were defined and an optimal set of kinetic parameters computed (Fig 3C and 3D, S3 Fig, S2 Table).

Because some of the observables were estimated from published datasets, and individual parameter estimates are each based on a small number of observables, it is possible that some parameter estimates are off and may lead to a wrong conclusion. To account for that possibility, we restricted our analysis to population statistics only. Additionally, we performed a robustness analysis by generating alternative datasets by adding noise to the observables and examined how often we reached the main conclusion (S3 Table, S1 Text).

### Dynamics of naive T-cell turnover according to modeling

**Fig 4:**
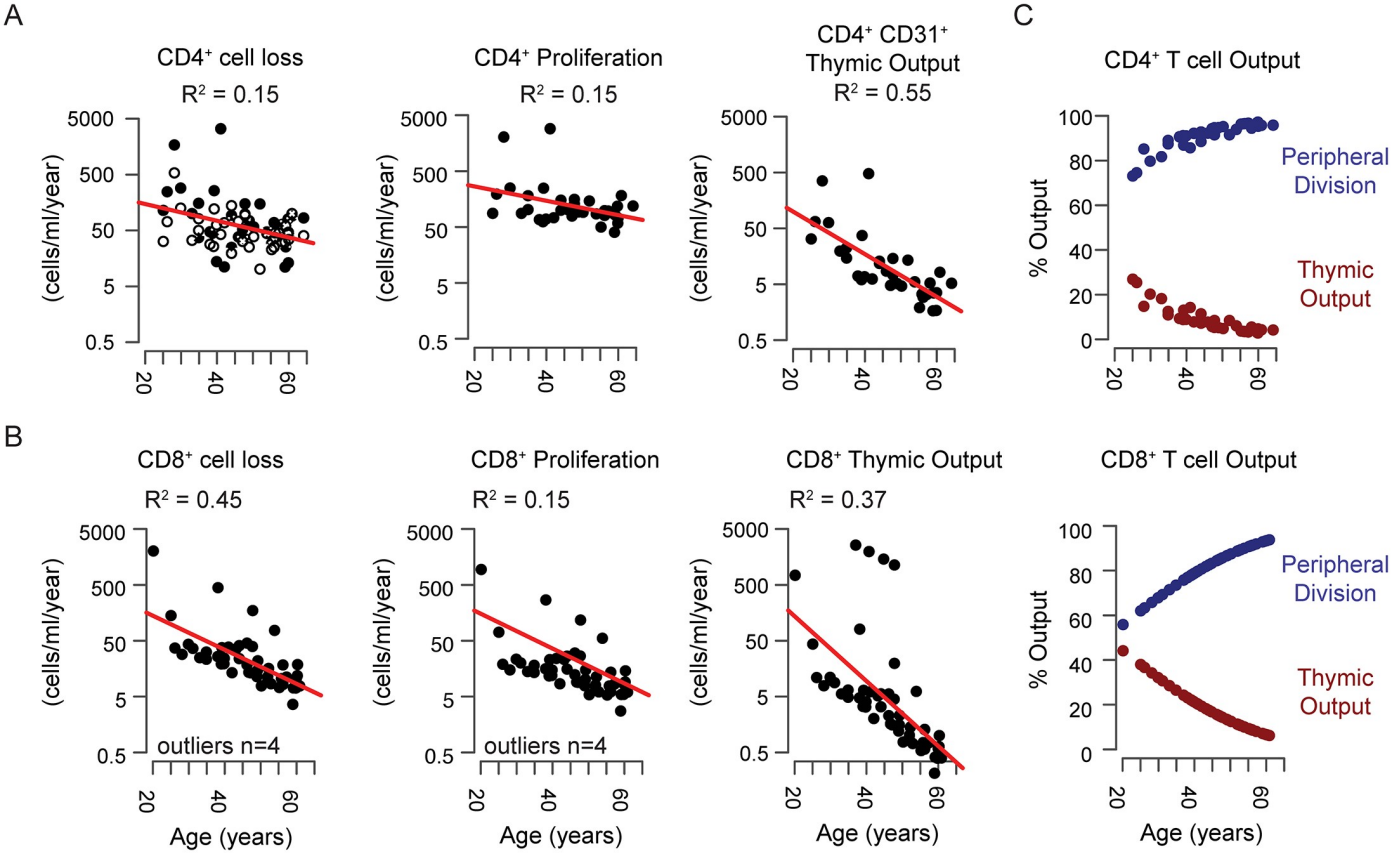
The CD4^+^ and CD8^+^ naive T-cell populations exhibit reduced cell loss, peripheral division, and thymic production throughout adulthood. (A) Dynamic rates of cell loss, homeostatic turnover and thymic activity for CD4^+^ T cells based on model II+ (Fig 3D). For cell loss CD4^+^CD31^+^ loss rates are shown as black dots and CD4^+^CD31^−^ cells are shown as white dots. Only CD4^+^CD31^+^ naive T cells are subject to homeostatic turnover and thymic production according to model II+, and thus CD4^+^CD31^−^ cells are not depicted. (B) Dynamics of CD8^+^ naive T-cell loss, proliferation, and thymic activity relative to donor age. (C) Relative contribution of thymic activity and peripheral turnover for CD4^+^ (top) and CD8^+^ (bottom) naive T-cell populations expressed as %Output or fraction of new cells contributed by each mechanism.

Cell number can be maintained by thymic output, peripheral proliferation, and/or lower cell loss. Our calculations suggest that total thymic output declines exponentially with age. Loss rates decline for both CD4^+^ and CD8^+^ naive T-cell populations relative to age but did not differ significantly between cell types (Fig 4A and 4B, Wilcoxon rank sum test, *p* > 0.05). Proliferative activity was significantly higher in the CD4^+^ population (Wilcoxon rank sum test, *p* < 1 × 10^−6^). Although the net proliferative output in the CD4^+^ and CD8^+^ naive T-cell populations declines with donor age, the contribution to cell number maintenance increases, from around 60% at 20 years of age to more than 95% at 65 years of age, owing to a substantial decline in thymic output over this timeframe. Total thymic output in the CD4^+^ naive T-cell population is twice that of the CD8^+^ naive T-cell population (Fig 4A and 4B, Wilcoxon rank sum test, *p* = 0.002). However, the relative contribution of thymic output to the peripheral CD8+ naive T-cell population is slightly higher because of the numerical differences in peripheral proliferative output (Fig 4A and 4C, Wilcoxon rank sum test, *p* = 0.04).

A common explanation for how total CD4^+^ naive T-cell numbers persist throughout adulthood relative to CD8^+^ naive T cells rests primarily on their ability to expand as they differentiate into CD4^+^CD31^−^ naive T cells [12, 38]. Our data on average cell DNA age provide the first estimate of the rate at which this transition occurs, as we see that CD4^+^CD31^+^ cells exhibit increased average cellular DNA age and life spans in older donors (Figs 1D and 3D), indicating that CD31 may be better treated as a marker of limited CD4^+^ naive T-cell peripheral expansion rather than recent thymic egress.

### Modeling the relationships between CD4^+^CD31^+^ and CD4^+^CD31^−^ naive T cells suggests CD31^−^ T cells are terminally expanded

Little is known about the mechanisms leading to loss of CD31 expression or about the consequences of CD31 down-regulation in the human CD4^+^ naive T-cell subset [39, 40]. It is clear that a similar process does not occur in CD8^+^ naive T cells or in mouse models. CD4^+^CD31^−^ naive T cells accumulate throughout adult life and exhibit decreased TREC content, potentially indicating that peripheral expansion of CD4^+^ naive T cells coupled with loss of CD31 expression maintains a steady state in CD4^+^ naive T-cell numbers throughout adulthood [18, 40, 41]. As a population, CD8^+^ naive T cells contain TREC content comparable to that of CD4^+^CD31^+^ naive T cells, and the number of CD8^+^ naive T cells declines with age [8, 13, 23].

Because CD4^+^CD31^−^ naive T cells have decreased TREC content and accumulate over time, we anticipated that this population would have a higher rate of turnover and hence a shorter life span than CD4^+^CD31^+^ cells. Our results, however, indicate that the two populations age comparably (Fig 1D, S2 Table). Moreover, the age-related increase in CD4^+^CD31^+^ naive T-cell life span suggests that CD31 expression is not indicative of recent thymic production but rather reflects peripheral division history, as CD31^+^ naive T cells in 50–64-year-old individuals had average cell DNA ages of 4–11 years (Fig 1D). We therefore decided to test several hypotheses concerning the dynamic behavior of each population of CD4^+^ naive T cells using our linear models (Fig 3C and 3D, S3 Fig, S2 Table, S1 Text). In particular, we wanted to assess whether our data were consistent with the possibility that CD31^−^ naive T cells represent a terminally expanded population of naive CD4^+^ T cells constantly fed by homeostatically dividing CD4^+^CD31^+^ naive T cells throughout life.

We addressed this hypothesis by setting the peripheral division rates of CD4^+^CD31^−^ naive T cells to 0 in our linear models (H2). Additional hypotheses were that death rates of CD31^+^ and CD31^−^ cells were equal (H1) and that CD31^+^ naive T cells do not undergo homeostatic division (H3), in each case resulting in removal of a single dynamic variable (S3 Fig, S2 Table, S1 Text). We determined goodness of fit by measuring the sum of squared errors (SSE) and the differences in Akaike information criterion values (ΔAICc) as a measure of model accuracy [42]. The scenario in which CD31^−^ naive T cells were assumed to be a nondividing population (scenario II) provided a better potential fit based on the ΔAICc, compared to our initial model (scenario IV, no assumptions), though each had roughly equivalent SSE (ΔAICc = 2.3, S3C Fig). The evidence ratio was exp(2.3/2) = 3.2, suggesting that scenario IV remains plausible. Robustness analysis (S3 Table) confirms this analysis; using perturbed datasets, scenario IV was selected more frequently as noise levels were increased. Our initial model (scenario IV) indicated an age-related decline in CD31^−^ naive T-cell proliferation but not CD31^+^ naive T-cell proliferation, consistent with the idea that CD31+ naive T cells have heightened homeostatic proliferative potential. Thus, our data are consistent with the hypothesis that CD4^+^CD31^−^ naive T cells do not undergo peripheral homeostatic division. Notably, our initial model (scenario IV) indicated an age-related decline in CD31^−^ naive T-cell proliferation but not in CD31^+^ naive T-cell proliferation, consistent with the idea that CD31^+^ naive T cells have heightened homeostatic proliferative potential (Fig 3D, S3 Fig).

### CD31 expression on CD4^+^ naive T cells is linked to homoeostatic proliferative potential in vivo

**Fig 5:**
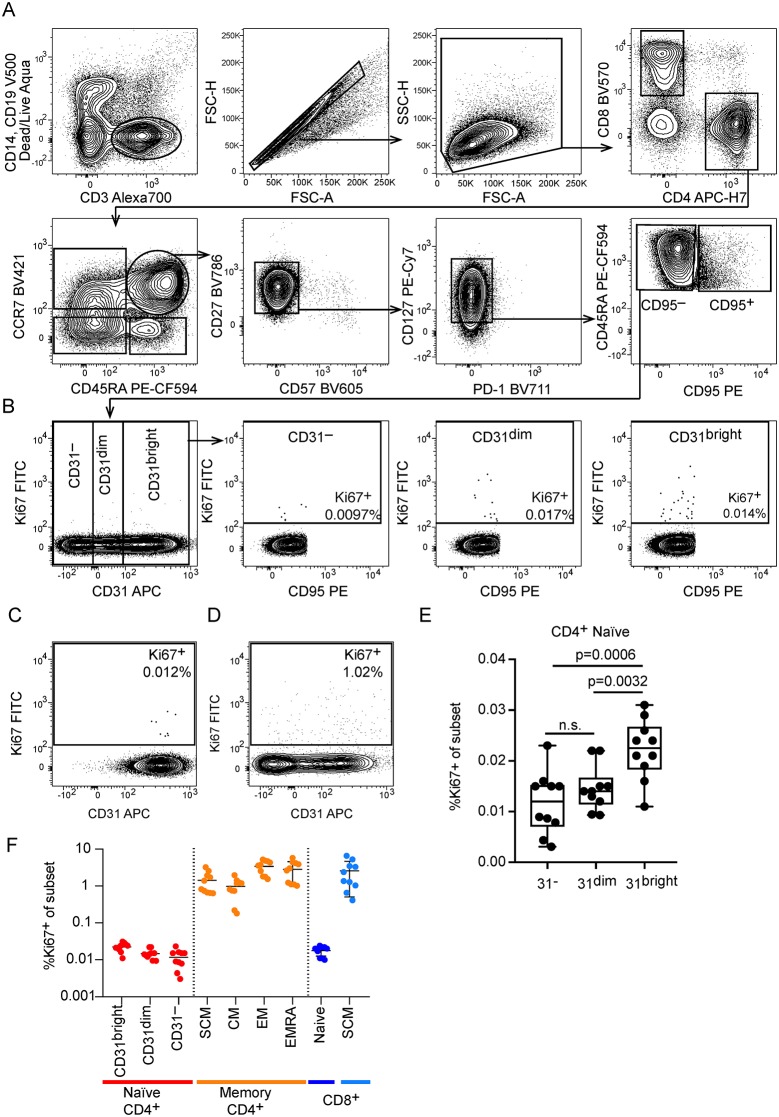
CD4^+^CD31^+^ naive T cells proliferate more than CD4^+^CD31^−^ naive T cells in vivo. (A) Gating strategy to identify naive T cells. Naive CD4^+^ T cells were identified as live, CD14^−^CD19^−^CD3^+^CD4^+^CD8^−^CD45RA^+^CCR7^+^CD57^−^CD27^+^PD1^−^CD127^+^CD95^−^ cells to exclude potential contaminating populations of activated cells including SCM cells. The same strategy was used to identify CD8^+^ naive T cells by switching to the CD4^−^CD8^+^ fraction. (B) Representative example of CD4^+^ naive T-cell distribution for CD31 expression and gating strategy to define CD4^+^CD31^bright^, CD4^+^CD31^dim^, and CD4^+^CD31^−^ cells. (C) Frequency of Ki67^+^ dividing cells in the CD8^+^ naive T-cell gate. (D) Frequency of Ki67+ dividing cells within the SCM population present in the conventional CD4^+^CD45RA^+^CCR7^+^ gate. (E) Summary of all donors showing frequency of Ki67^+^ dividing cells for each population identified with our gating strategy. Significantly fewer Ki67^+^ cells were observed in the CD4^+^CD31^−^ naive T-cell fraction as compared to the CD31^bright^ population (*p* = 0.0006, paired *t* test) and the CD31^dim^ versus CD31^bright^ population (*p* = 0.0032, paired *t* test). (F) Frequency of Ki67^+^ cells in CD4^+^ naive T cells, CD4^+^ memory T-cell subsets, and in CD8^+^ naive and CD8^+^ SCM subsets for all donors. APC, allophycocyanin; CCR7, C-C chemokine receptor 7; CM, central memory; EM, effector memory; EMRA, terminal differentiated effector memory (CD45RA^+^); FITC, fluorescein isothiocyanate; FSC-A, forward scatter area; FSC-H, forward scatter height; n.s., not significant; PD-1, programmed cell death protein 1; PE, phycoerythrin; SCM, stem cell memory; SSC-H, side scatter height.

Two previous studies have reported that CD4^+^CD31^−^ naive T cells may have limited proliferative potential to IL-7 in vitro [43, 44]. It remains unclear, however, whether differences exist for each population in vivo, and no attempts have been made, to our knowledge, to measure the proliferation of each population using markers that exclude contaminating memory populations in the naive T-cell pool. We therefore profiled 11 healthy adult donors (average age 48 ± 16 years, range 25–69) using a comprehensive panel of markers specific to naive T cells (CD4^+^CD45RA^+^CCR7^+^CD27^+^CD127^+^CD57^−^PD1^−^CD95^−^) and the proliferation marker Ki67, which labels cells that underwent mitosis within the preceding 72 hours [45], to define the frequency of dividing naive T cells in relation to CD31 expression in vivo (Fig 5A and 5C, S1 Data). Using these stringent criteria to focus on truly naive T cells, we observed there were very few naive T cells exhibiting evidence of recent proliferation (0.003%–0.031%), as expected (Fig 5B, 5C, 5E and 5F). By contrast, Tscm cells (CD4^+^CD45RA^+^CCR7^+^CD27^+^CD127^+^CD57^−^PD1^−^CD95^+^), which have a similar phenotype as CD4^+^ naive T cells, apart from expression of CD95, exhibited much higher frequencies of dividing cells, again indicating that this population may impact turnover estimates in prior studies (Fig 5D). We noted that CD31 expression within the CD4^+^ naive T-cell population is better viewed as a continuum rather than as discrete CD31^+^ and CD31^−^ populations (Fig 5B). Therefore, we gated on CD31^bright^, CD31^dim^, and CD31^−^ populations and quantified Ki67 frequencies in each (Fig 5B and 5E). We noted that the CD31^−^ population showed significantly less proliferation across all donors (0.00204%–0.023%, mean: 0.01%, SD: 0.006%), providing additional support for our hypothesis that these cells exhibit limited peripheral turnover potential (Fig 5E). CD8^+^ naive T cells had high CD31 expression and comparable frequencies of dividing cells to CD4^+^CD31^+^ naive T cells in all donors (range 0.0099%–0.024%, mean: 0.017%, SD: 0.005%) (Fig 5C and 5F).

### Single-cell proteomic analysis of CD4^+^ naive T cells reveals a link between CD31 expression and basal NF-κB phosphorylation

We reasoned that we could potentially examine molecular pathways regulating homeostatic proliferation by examining CD4^+^ naive T cells relative to CD31 expression. To this end, we utilized mass cytometry to interrogate differences in both phenotypic and functional properties of single naive T cells that correlated with differences in CD31 expression level. This allowed us to more accurately address differences according to the range of CD31 expression detected within the total CD4^+^ naive T-cell pool (Fig 5B).

**Fig 6:**
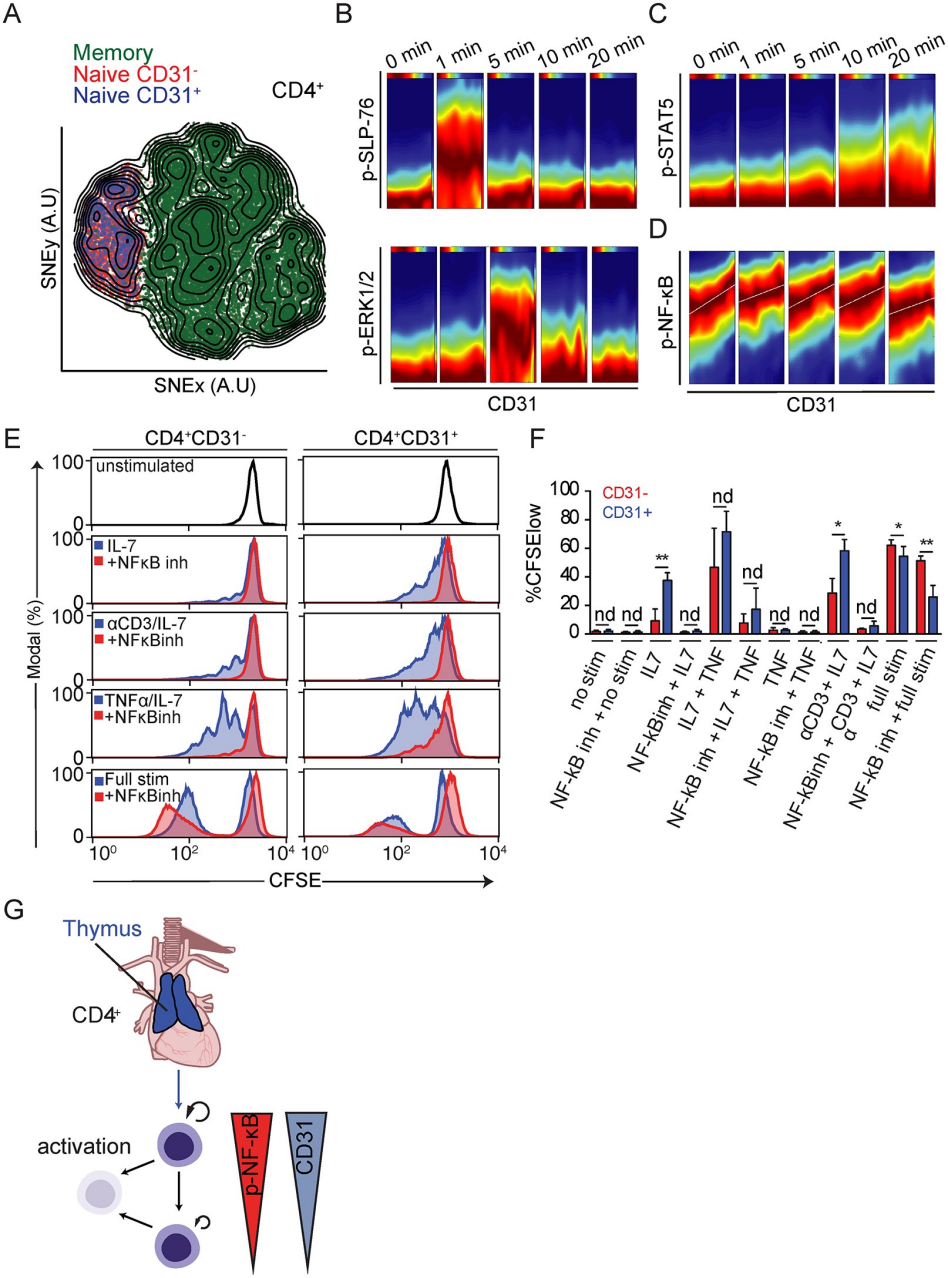
Evidence for a link between replicative history and homeostatic proliferation potential controlled by NF-κB. (A) Memory/effector (green) and naive CD4^+^ T cells divided into CD31^−^ (red) and CD31^+^ (blue) cells were merged and their high-dimensional single-cell phenotypes analyzed by mass cytometry visualized using a tSNE dimensionality reduction. (B) DREVI plots depicting correlation between CD31 and p-SLP-76, p-ERK1/2, (C) p-STAT5, and (D) p-NF-κB throughout a 20-minute homeostatic stimulation with αCD3 and IL-7. Data are representative of four separate experiments on eight donors. (E) Proliferation of sort-purified CD4^+^CD31^−^ (left) and CD4^+^CD31^+^ (right) naive T cells in response to different stimulation conditions (blue histograms) as well as in the presence of the NF-κB inhibitor 6-amino-4–4-phenoxyphenylethylamino-quinazoline (red histograms). Data represent four experiments on eight donors. (F) Summary data from four independent donors showing percent CFSE^low^ (proliferated) cells within the CD31^+^ and CD31^−^ fraction of naive CD4^+^ T cells relative to different stimulatory conditions. Cells were treated with vehicle (DMSO) or NF-κB inhibitor (“NFκBinh” 6-amino-4–4-phenoxyphenylethylamino-quinazoline) for each stimulation condition (S3 Data). Full Stim: αCD3/αCD28/IL-2. (G) Schematic summary of the dynamics of CD4^+^ naive T-cell homeostasis in adult humans. A.U., arbitrary units; CFSE, carboxyfluorescein succinimidyl ester; DREVI, conditional-density rescaled visualization; IL, interleukin; nd, no difference; NF-κB, nuclear factor κB; p-ERK1/2, phosphorylation of extracellular signal–regulated kinase 1/2; p-NF-κB, phosphorylation of NF-κB; p-SLP-76, phosphorylation of SH2 domain-containing leukocyte protein of 76 kilodaltons; p-STAT5, phosphorylation of signal transducer and activator of transcription 5; stim, stimulation; TNF, tumor necrosis factor; tSNE, t-stochastic neighborhood embedding.

We first confirmed that CD45RA^+^CCR7^+^CD4^+^CD31^+^ and CD45RA^+^CCR7^+^CD4^+^CD31^−^ naive T cells were phenotypically indistinguishable with respect to 14 additional T-cell activation and differentiation markers including the homeostatic cytokine receptor IL-7R (S5 Table) visualized using t-stochastic neighborhood embedding (t-SNE) and ACCENSE (Fig 6A) [46, 47]. We did not exclude Tscm from these experiments, as we did not have suitable markers in our CyTOF antibody panel to identify these cells. Next, we performed in vitro stimulation assays on total peripheral blood mononuclear cells (PBMCs) and monitored signaling states of individual cells by mass cytometry with antibodies targeting phospho-proteins involved in T-cell activation pathways (extracellular signal–regulated kinase [ERK 1/2] and SH2 domain-containing leukocyte protein of 76 kilodaltons [SLP-76]) [48]. Temporal changes in the activation of different signaling pathways were directly related to differences in CD31 expression using standard Pearson’s correlations and a recent conditional-density estimator called conditional-density rescaled visualization (DREVI) [48]. With DREVI, the density estimate of the response variable is renormalized to obtain a conditional-density estimate of this variable given the abundance of another variable—CD31 cell surface expression, in this case. We observed the expected kinetic changes in phosphorylation of canonical signaling molecules SLP-76 and ERK upon T-cell receptor cross-linking, but these patterns showed no relationship with CD31 expression (Fig 6B).

We addressed whether differences in CD31 expression levels correlated with differential responses to the naive T-cell growth factor IL-7. Because T-cell receptor engagement has been implicated as an important component of homeostatic signaling of naive T cells in mice, we also included T-cell receptor cross-linking with antibodies against CD3 in our assays [49]. We observed that inclusion of CD3 cross-linking resulted in only a modest increase in naive T-cell proliferation and evidence of a small degree of T-cell activation, as indicated by loss of CD45RA expression (S4A Fig). The proximal signaling event downstream of IL-7R stimulation is the phosphorylation of signal transducer and activator of transcription 5 (STAT5). We observed that phosphorylation of STAT5 in response to IL-7 occurred within 10 minutes after stimulation and increased throughout the course of stimulation. STAT5 phosphorylation was found to be equivalent across the CD4^+^ naive T-cell population regardless of CD31 expression (Fig 6C, S5 Fig). This observation was consistent with the fact that IL-7R levels were equivalent for naive CD4^+^CD31^+^ and CD31^−^ cells (Fig 6A).

A comprehensive examination of all changes in measured phospho-protein expression relative to CD31 expression in naive CD4^+^ T cells revealed a consistent positive relationship between CD31 expression and phosphorylation of the NF-κB subunit p65 (RelA) (Fig 6D, S5 Fig, S5 Table, S2 Data). This relationship was observed in the absence of stimulation as well as in the presence of IL-7 and CD3 cross-linking. Stimulation with tumor necrosis factor (TNF), which is known to act through NF-κB, resulted in a slight increase in NF-κB phosphorylation across the naive CD4^+^ T-cell population regardless of CD31 expression (S5 Fig). Interestingly, basal NF-κB phosphorylation has been shown to play an important role in determining naive T-cell survival in rodents [50, 51], prompting us to investigate this pathway further.

### Homeostatic proliferation in response to IL-7 is dependent upon NF-κB signaling

We next determined whether CD4^+^CD31^+^ and CD4^+^CD31^−^ naive T cells showed differences in proliferative potential in vitro in response to IL-7 stimulation using carboxyfluorescein succinimidyl ester (CFSE) dilution assays. For these experiments, we used the same purification protocols used for ^14^C measurements, which do not remove contaminating Tscm from the naive T-cell fraction. Consistent with prior reports, we noted a significant reduction in proliferation in response to IL-7 stimulation by CD4^+^CD31^−^ naive T cells relative to CD4^+^CD31^+^ naive T cells (Fig 6E). Both populations proliferated and underwent differentiation in response to T-cell activation stimuli, with CD4^+^CD31^−^ naive T cells exhibiting a small but statistically significant increase in proliferation. Interestingly, CD8^+^ naive T cells were highly responsive to IL-7 stimulation in vitro despite exhibiting generally lower turnover rates in vivo, as measured by ^14^C levels.

To address whether differences in basal NF-κB phosphorylation levels account for differential proliferative potential of CD4^+^CD31^+^ and CD4^+^CD31^−^ naive T cells, we measured homeostatic proliferation of each population in the presence of an NF-κB inhibitor (6-amino-4–4-phenoxyphenylethylamino-quinazoline). Inhibition of NF-κB completely abolished homeostatic proliferation but did not completely inhibit proliferation in response to activation signals (Fig 6E and 6F) [43]. Additionally, we found that we could rescue homeostatic proliferation of the nonresponsive CD4^+^CD31^−^ naive T cells by promoting NF-κB phosphorylation through the addition of TNF (Fig 6E and 6F). Notably, proliferation in the presence of TNF was still dependent upon IL-7 and did not result in activation of naive T cells as determined by CD45RA down-regulation (Fig 6F, S4A Fig). We observed a similar dependence on NF-κB activity for homeostatic proliferation of CD8^+^ naive T cells, which in general showed heightened responses to IL-7 stimulation (S4B and S4C Fig). Thus, our data support a model in which gradual loss of CD31 expression by naive CD4^+^ T cells is associated with decreased NF-κB phosphorylation, leading to reduced peripheral homeostatic proliferative potential but comparable activation potential (Fig 6G).

## Discussion

Here, we provide a comprehensive dataset defining the global turnover rates of the naive T-cell pool in adult human beings until 65 years of age. Our findings extend nicely upon estimates made concerning the dynamic regulation of the generation of the naive T-cell pool during the first 20 years of life [5]. Our conclusions were not wholly consistent, however, with a recent study tracking naive T-cell turnover in donors between 65 and 75 years of age, for which no age-related changes in turnover were documented relative to younger donors [16]. This discrepancy may reflect the fact that individuals over 65 years of age contain elevated frequencies of memory cells and activated T cells within the population traditionally identified as naive T cells, as well as changes that are reported to occur in advanced age that lead to collapse of naive T-cell diversity [11, 52, 53]. Additional efforts to define the population-level turnover rates of naive T cells in healthy humans over 65 years of age will be needed to better address these differences. Indeed, we observed variability in the cell DNA age estimates in our 50–65-year-old donors, which could reflect donor-dependent differences in naive T-cell homeostasis, perhaps contributing to changes in naive T-cell homeostasis reported in a fraction of elderly individuals [11].

It is now recognized that the human naive T-cell pool fundamentally differs from laboratory mice, with respect to factors responsible for maintaining cell numbers and diversity throughout life [8]. Mice maintained in pathogen-free environments typically live for 2 to 3 years, throughout which time the thymus accounts for nearly all new production of naive T cells [54]. In humans, the thymus is capable of generating hundreds of millions of new naive T cells every year until roughly 30 years of age, as predicted by our models as well as previously published estimates [8]. Histological data support this conclusion, as medullary and cortical regions in which T-cell development occurs are visible until roughly 40 years of age [4, 7]. The loss of thymic function after 40 years has prompted speculation that sudden increases in human life spans have outpaced evolutionary processes needed to maintain an adaptive immune system in older age [55]. Nonetheless, we maintain a diverse naive repertoire for several decades beyond the time that the thymus ceases to function, primarily because of peripheral homeostatic division of existing naive T cells.

Sustaining a highly diverse naive T-cell repertoire solely through replication of existing clones presents an interesting challenge. Naive T cells compete for resources and space in tissues, which might lead to certain clones, particularly those made early in life, outcompeting unexpanded, newly produced clones later in life [35]. This amounts to a cellular equivalent of the “tragedy of the commons,” which posits that individuals acting in their own self-interest will deplete a shared resource, bringing about the eventual collapse of the system [56]. This is particularly true for access to IL-7, which is necessary for naive T-cell survival and proliferation and is expressed at constant, low levels by stromal cells in the secondary lymphoid tissues [57]. The impact of IL-7 levels on naive T-cell turnover is confirmed by the observation that therapeutic administration of high levels of IL-7 promotes substantial increases in Ki67+ naive T cells after 1 week [58].

This prompts the question: How do naive T cells undergo peripheral expansion without leading to unequal clonal growth and collapse of diversity? The slow, constant turnover of the population, with estimated individual naive T-cell division rates on the order of once every 3–8 years, provides a general mechanism to support population diversity over 30–40 years after thymic involution. Additionally, we hypothesized that mechanisms are likely to exist to support equality in clonal growth across the diverse repertoire of naive T-cell clones and ensure that expanded clones do not consume space and resources at the expense of newly generated or unexpanded clones.

We provide evidence of such a mechanism by monitoring cell DNA age changes in the CD4^+^ naive T-cell population relative to subpopulations with different replicative histories. We observed that expanded populations of CD4^+^CD31^−^ naive T cells are less likely to undergo homeostatic proliferation in the periphery as compared to less expanded CD4^+^CD31^+^ naive T cells. Mechanistically, we show that this is partly regulated by changes in basal NF-κB activity that alter downstream effects of IL-7 stimulation, leading to restrictions on proliferation in response to the prototypical homeostatic growth factor important for naive T-cell survival and expansion. Such a mechanism could potentially favor proliferation of clones in the less expanded CD4^+^CD31^+^ compartment, facilitating even expansion of all naive T-cell clones in the periphery.

Our proposed mechanistic link between clone size and capacity to compete for growth factors, such as IL-7, may not be unique to naive T cells or even to lymphocytes, for that matter. The existence of unique, heritable antigen receptors allows one to unambiguously classify a T or B cell according to the specificity that is conferred upon each clone by these receptors. However, many cell types may exhibit clonal differences that could reflect functional diversity within a complex multicellular organism. As populations of cells all require shared growth factors, it is intriguing to speculate whether decreasing fitness relative to clonal size, as it relates to competition for survival signals, is a general mechanism in biological systems to sustain high levels of clonal diversity with aging, particularly in cells in which loss of telomere length is not a limiting factor [59, 60].

Interestingly, CD8^+^ naive T cells appear to exhibit reduced homeostatic proliferation in vivo relative to CD4^+^ naive T cells. However, in vitro, we observed that CD8^+^ naive T cells responded equivalently, if not more than, CD4^+^CD31^+^ naive T cells to IL-7, suggesting that additional factors contribute to the regulated, slow rate of homeostatic division of CD8^+^ naive T cells in vivo. A simple explanation would again be that IL-7 itself is highly limiting in vivo, as exogenous IL-7 therapy led to a massive increase in Ki67^+^CD8^+^ naive T cells (>50% of the population) within a week after administration [58].

An interesting implication of our findings is how inflammation could influence diversity within the naive T-cell pool. We demonstrate that addition of TNF, a factor commonly increased in inflammatory settings, allows for extensive proliferation of all naive T-cell subsets to IL-7, including expanded CD4^+^CD31^−^ naive T cells, which typically show minimal proliferation to IL-7 in vitro. Chronic inflammatory conditions such as rheumatoid arthritis are known to significantly alter the naive T-cell repertoire in humans [61, 62]. Moreover, the well-described collapse in diversity seen in old age may be a direct consequence of increased chronic inflammation, termed “inflammaging,” which is increasingly recognized as a major contributor to age-related immune dysfunction among other diseases [63, 64]. Given that there are approved therapies for TNF inhibitors in the context of inflammatory disorders, it could be interesting to explore how these may influence naive T-cell diversity in elderly individuals [65]. Moreover, defining the factors that influence changes in naive T-cell diversity and the consequences of these changes in the elderly will be of increasing importance as human life spans continue to increase in the coming decades.

## Materials and methods

### Ethics statement

Buffy coats were obtained from anonymous healthy blood donors at Blodcentralen, Karolinska University Hospital, with the approval of the ethical review board in Stockholm, Sweden (2006/229-31/3). All donors are anonymous, and no genetic information is used in the manuscript.

### Isolation of cells

Red blood cell lysis was performed by combining the total buffy coat with a (1:3) solution of red blood cell lysis buffer (155 mM NH_4_CL, 12 mM NaHCO_3_, 0.1 mM EDTA) and incubated for 10 minutes at room temperature. After the 10-minute incubation, the samples were centrifuged in 50-mL conical tubes and resuspended in 5 ml PBS (containing 2 mM EDTA) to which 45 ml of red blood cell lysis buffer was again added followed immediately by a second centrifugation step. The remaining cells were washed two times in MACS buffer (PBS, 0.5% bovine serum albumin, 2 mM EDTA) to remove residual lysis buffer. Samples were filtered through 40-μM mesh filters.

### Magnetic bead purification

Total white blood cells were obtained by red blood cell lysis. An antibody cocktail designed for negative selection of purified naive T cells (naive pan T-cell purification kit, Miltenyi Biotec) was used together with extra αCD15/CD14 beads. This allowed for a single selection step over D columns that gave highly purified CD4^+^ and CD8^+^ T naive T cells (typically >90%) prior to FACS.

### Flow cytometry and cell sorting

Enriched populations of cells obtained after magnetic selection protocols were incubated with a cocktail of antibodies and sorted by FACS: CD3ε-FITC (clone UCHT1, BD Biosciences), CD4-APC Cy7 (clone RPA-T4, BD Biosciences), CD8α-BV768 (clone RPA-T8, BD Biosciences), CD45RA-PE-CF594 (clone HI100, BD Biosciences), CCR7-BV450 (clone 150503, BD Biosciences), and CD31-APC (clone AC128, Miltenyi Biotec). Cells were labeled in MACS buffer containing antibodies at 4 °C for 30 minutes and washed extensively and filtered to remove dead cells and debris prior to sorting. FACS was performed using a BD Influx cell sorter equipped with blue (480 nm), red (640 nm), and violet (405 nm) lasers. Sort purities were determined after sorting for all populations.

### Flow cytometry analysis of Ki67 expression and Tscm frequency

For analysis of Ki67 expression in T-cell subsets, we thawed cryopreserved PBMC and stained them with CD3ε-Alexa700 (clone UCHT1), CD4-APC-H7 (clone SK3), CCR7-BV421 (clone 150503), CD27-BV786 (clone L128), CD49F-BV650 (clone GoH3), CD57-FITC (clone NK-1), CD14-V500 (clone MΦP9), CD19-V500 (clone HIB19) (all from BD Biosciences), CD127-PE-Cy7 (clone R34.34, Beckman Coulter), CD8α-BV570 (clone RPA-T8, BioLegend), CD95-PE (clone DX2, BioLegend), CD45RA-PE-Texas Red (clone MEM-56, ThermoFisher), CD31-APC (clone AC128, Miltenyi Biotec), and LIVE/DEAD fixable Aqua dead cell stain (ThermoFisher) in PBS containing 2% fetal bovine serum (FBS) and 2 mM EDTA. After washing, the PBMCs were fixed with fixation/permeabilization buffer (Transcription Factor Staining Buffer, Ebioscience) for 30 minutes at 4 °C, washed twice with permeabilization buffer (Transcription Factor Staining Buffer, Ebioscience), and stained for 30 minutes with anti-Ki67-FITC (clone 35/Ki-67, BD Biosciences) diluted in permeabilization buffer. Data were acquired on a BD Biosciences LSR Fortessa instrument equipped with 405-nm, 488-nm, 561-nm, and 647-nm lasers and analyzed using FlowJo software (v9.7.8). Naive CD4^+^ and CD8^+^ T cells were defined as CCR7^+^CD45RA^+^ CD95^−^ cells, and Tscm cells were defined as CCR7^+^CD45RA^+^CD95^+^ cells, among CD3^+^CD4^+^ or CD3^+^CD8^+^ live, CD14^−^CD19^−^ lymphocytes.

### Analysis of phospho-NF-κB by flow cytometry

Peripheral blood was collected in heparin tubes, and PBMCs were isolated using density centrifugation. The freshly isolated PBMCs were stained with antibodies against CD31 Alexa 647 (Clone WM59, BioLegend), CD14 V500 (clone MfP9, BD Biosciences), CD19 V500 (clone HIB19, BD Biosciences), CCR7 BV421 (clone 150503, BD Biosciences), CD8α BV570 (BioLegend), and LIVE/DEAD fixable dead cell stain near IR (Invitrogen) in RPMI1640 with 10% FCS (R10 medium) for 30 minutes at 4 °C. After washing twice, 50 ml (10^6^ PBMCs) was added to 150 ml prewarmed (37 °C) media with or without 15 ng/ml TNF and incubated at 37 °C for 3 minutes, after which 200 ml prewarmed 4% paraformaldehyde was added. The cells were fixed for 20 minutes at 37 °C and for an additional 30 minutes at 4 °C, washed twice in PBS containing 2 mM EDTA and 1% FCS (FACS wash), and resuspended in 5 ml FACS wash. Subsequently, 200 ml methanol (−20 °C) was added, and the cells were incubated at −20 °C for 3 hours, washed twice in FACS wash, and incubated with the following antibodies for 1 hour at room temperature: CD4 FITC (clone SK3, BD Biosciences), CD3 Alexa 700 (clone UCHT1, BD Biosciences), CD45RA BV786 (clone HI100, BD Biosciences), and pNF-κB S529 PE-CF594 (clone K10-895.12.50, BD Biosciences). The cells were washed twice and immediately acquired on an LSR Fortessa (BD Biosciences) and analyzed using FlowJo v9.8 (Treestar). Naive CD4+ T cells were defined as CD45RA^+^CCR7^+^ among single live, CD14^−^CD19^−^CD3^+^CD4^+^ lymphocytes.

### DNA isolation

The DNA extractions were done in an ISO 8 cleanroom to reduce carbon contamination. Beforehand, the glass utensils were baked at 450 °C for 4 hours. To each sample, 1 ml of lysis buffer (100 mM Tris [pH 8.0], 200 mM NaCl, 1% SDS, and 5 mM EDTA) and 12 μl of Proteinase K (40 mg/ml) were added. The sample was then incubated overnight at 65 °C. RNase cocktail (6 μl, Ambion) was added to each sample, and the samples were then incubated at 65 °C for 1 hours. Each sample received half a volume of NaCl (5 M). After a 30-second vortex, the tubes were centrifuged at 13,000 rpm for 6 minutes. The pellet was discarded and the supernatant transferred to a glass tube with 3 volumes of absolute ethanol. The tubes were gently agitated for 30 seconds. The DNA pellet formed in the previous step was washed three times in buffer (70% Ethanol [v/v] and 0.5 M NaCl) and later dried at 65 °C overnight to evaporate all the ethanol. DNase/RNAase free water (500 ml, GIBCO/Invitrogen) was added to each vial and left overnight at 65 °C to resuspended the DNA. The concentration and purity were assessed by UV spectroscopy (NanoDrop).

### Accelerator mass spectrometry

A special sample preparation method has been developed for the μg-sized DNA samples [29]. The purified DNA samples were received suspended in water. The samples were subsequently lyophilized to dryness under vacuum and centrifugation. Excess CuO was added to the dried samples in quartz tubes, which were then evacuated and sealed with a high-temperature torch. The quartz tubes were placed in a furnace set at 900 °C for 3.5 hours to combust all carbon to CO_2_. The evolved gas was cryogenically purified and trapped. The CO_2_ gas was converted to graphite in individual sub-mL reactors at 550 °C for 6 hours in the presence of zinc powder as reducing agent and iron powder as catalyst. The graphite targets were measured at the Department of Physics and Astronomy, Ion Physics, Uppsala University, using a 5-MV Pelletron tandem accelerator. Stringent and thorough laboratory practice is necessary to minimize the introduction of stray carbon into the samples, including preheating of all glassware and chemicals prior to samples preparation. Large CO_2_ samples (>100 μg) were split, and δ^13^C was measured by stable isotope ratio mass spectrometry, which established the δ^13^C correction to −24.1‰ ± 1‰ (2 SD) for leucocyte samples. Corrections and reduction of background contamination introduced during sample preparation were made as described previously [29]. The measurement error was determined for each sample and ranged between ±8‰ and 24‰ (2 SD) Δ^14^C for the large sample and small samples (10 μgC), respectively. All ^14^C data are reported as decay-corrected Δ^14^C or fraction modern. All accelerator mass spectrometry analyses were performed blind to age and origin of the sample.

### Mass cytometry and T-cell activation assays

Total PBMCs were purified from buffy coats by density centrifugation (FH). PBMCs were washed and subsequently resuspended in cold cell culture medium (RPMI 10% FBS, L-Glu). The PBMCs were divided into separate tubes and incubated for 30 minutes on ice with biotinylated αCD3 (1 μg/ml, OKT3; Miltenyi), αCD3/CD28 (CD28, 10 μg/ml, 15E8; Miltenyi)/αCD2 (10 μg/ml, LT2; Miltenyi), or nothing (nonstimulated control). All groups were incubated with αCD31 coupled to 148Nd prior to stimulation, for staining (not cross-linking), and initial experiments performed without preincubation of αCD31 gave comparable results for all measures, albeit with reduced resolution of CD31 expression (data not shown). The cells were washed one time with an excess of cell culture medium (100×), resuspended in prewarmed cell culture medium, and transferred to 96-well U-bottom culture plates containing prediluted cytokines (IL-2 or IL-7, both at 10 ng/ml final) and streptavidin (50 μg/ml) to cross-link simulation antibodies. Individual reactions were terminated by the addition of a formaldehyde-based fixation buffer (Cytodelics, Stockholm, Sweden) at different times after addition of cells and stored at 4 °C prior to staining and mass cytometry analysis. Cells were washed to remove fixation buffer and resuspended in staining buffer with a large panel of antibody markers (S5 Table). Cells were washed twice with staining buffer, filtered through a 35-μm nylon mesh filter followed by dilution to 5 × 10^5^ cells/ml. Cells were analyzed on a CyTOF2 mass cytometer (Fluidigm, South San Francisco, CA, USA) with software version 6.0.626 using a noise reduction with a lower convolution threshold of 200, a sigma value of 3, and an event length limitation of 10–150. FCS 3.0 files were exported without randomization and then normalized using internal bead standards. Cells were gated on as having an event length between 20 and 65 and being positive for iridium DNA-intercalator. Naive T-cell subsets were defined as lineage negative (CD11c, CD19, CD235a/b) and CD3^+^CD45RA^+^CCR7^+^CD27^+^ with CD4^+^ or CD8^+^ cells gated separately. Events were exported for downstream ACCENSE and DREVI analyses. The single-cell data were visualized by ACCENSE with CD4^+^CD31^+^ and CD31^−^ naive as well as memory/effector CD4^+^ T cells combined for dimensionality reduction. DREVI was performed on manually gated cell populations with naive defined as above and memory/effector cells defined as all non-naive T cells within the CD4^+^ T-cell fraction.

### In vitro T-cell proliferation assays

Naive T cells were purified as described for ^14^C dating protocols. The cells were subsequently washed in PBS and labeled with 5 μM CFSE (CellTrace, Invitrogen) in PBS for 10 minutes at 37 °C and washed extensively in cell culture medium. Labeled cells were added to 96-well U-bottom tissue-culture plates. Specified wells were precoated with αCD3 (HIT3A, BD Biosciences) by incubation with PBS containing αCD3 (0.1 μg/ml) at 37 °C for 4 hours and extensive washing to remove unbound antibody. Unstimulated cells were added to wells that had not been precoated with anti-CD3. Homeostatic proliferation assays were performed by culturing cells with recombinant human IL-7 (RND systems) at 10 ng/ml added on days 0, 2, 4, and 6 in the presence or absence of αCD3. Activation assays were performed by culturing cells with recombinant human IL-2 (10 ng/ml, RND Systems) in the presence of coated αCD3 and αCD28 (2 mg/ml, BD Biosciences) in the culture medium. For NF-κB inhibition assays, the inhibitor 6-amino-4–4-phenoxyphenylethylamino-quinazoline (Merck Millipore, Burlington, MA, USA) was reconstituted in DMSO at a stock concentration of 1 mM and administered at a final concentration of 10 nM on days 0 and 4. This concentration was chosen based on published ranges of toxicity for human T cells [51]. Control wells not receiving inhibitor received an equal volume of DMSO (1:100,000 dilution in culture medium) at the time inhibitor was added. Proliferation was measured on day 8 by labeling cells with standard memory/naive phenotyping antibodies used for naive purification (excluding CD3-FITC due to CFSE labeling) and performing flow cytometry analysis to measure CFSE dilution within naive (or memory) T-cell subsets (analysis done using FlowJo software version 9.2).

### Mathematical modeling

Statistical and mathematical analyses of data relating to ^14^C measurements are included as an attached supplemental text (S1 Text).

## Supporting information

S1 Fig Summary of donor characteristics and samples involved in ^14^C study. A complete overview is also provided in S1 Table. (A) Total number of samples included in ^14^C cell DNA age determination for Figs 1–4. (B) Distribution of samples relative to donor age (date of birth) for each sample set. (C) Collection date for each sample relative to donor age. Most samples were collected in 2014. (D) Carbon mass after isolation from purified DNA taken from each sample relative to donor age. (E) Frequency of CD31^+^ cells within the CD4^+^ naive T-cell fraction for all donors. (F) Summary of ^14^C measurements for all donors and cell types in study.
(TIFF)

S2 Fig Regressed data used for linear models presented used in Figs 3 and 4 to define dynamics of naive T-cell homeostasis. (A) CD8^+^ naive T-cell measurements for cell DNA age (based on this study), TREC content (taken from [23]), and cell numbers (taken from [13]). (B) CD4^+^ naive T-cell measurements for cell DNA age (based on this study), TREC content (taken from [8]), and cell numbers. Cell numbers were set to 500 cells/μl and defined in terms of CD31+ and CD31^−^ fractions based on regressed data from S1E Fig. TREC, T-cell receptor excision circle.
(TIFF)

S3 Fig Hypothesis testing for CD4^+^ naive T-cell dynamics using linear models relevant to Fig 3. (A) Schematic of CD4^+^ naive T-cell production, proliferation, differentiation, and activation/death. (B) Representation of calculated dynamic values for each scenario tested. (C) Table indicating different features of each scenario (Linear Models I–V) and SSE and ΔAICc (“dAICc”) for each scenario. Hypotheses tested for each scenario are listed below. ΔAICc; differences in Akaike information criterion values; SSE, sum of squared errors.
(TIFF)

S4 Fig Monitoring activation status of in vitro stimulated CD4^+^ and CD8^+^ naive T cells in CFSE assays from Fig 6. (A) CFSE dilution versus CD45RA expression for each stimulation condition. Minimal CD45RA down-regulation is observed with the exception αCD3 + IL-7 stimulation. Full stimulation (αCD3/αCD28 + IL-2) results in full activation of naive T cells. (B) CFSE dilution with different conditions for CD8^+^ naive T cells. Vehicle (DMSO—blue histograms) and treatment with NF-κB inhibitor (blue histogram) is shown. (C) Summary of four independent donors for CD8^+^ naive T cells. CFSE, carboxyfluorescein succinimidyl ester; IL, interleukin; NF-κB, nuclear factor κB.
(TIFF)

S5 Fig Extended data for Fig 6 showing raw correlations between CD31 and phosphor-proteins from CyTOF data and confirmation using flow cytometry. (A) Phosphorylation of STAT5 in unstimulated (“NS”) and in αCD3 + IL-7 (10 ng/mL) stimulated PBMCs. CD4+ naive T cells are identified by gating on lineage negative, CD3^+^CD4^+^CD45RA^+^CD27^+^CCR7^+^ and Pearson’s correlations are depicted for phosphor-STAT5 (y-axis) versus CD31 expression (x-axis). Values for different time points post-stimulation are shown (B) Phosphor-NF-κB (RelA/p65) versus CD31 expression on the same populations as in (A). (C) Confirmation of CyTOF results using flow cytometry to identify naive T cells (gate: Live, Lineage Negative, CD3^+^CD4^+^CD45RA^+^CCR7^+^) and monitoring phosphor-NF-κB (RelA/p65) (y-axis) versus CD31 expression (x-axis) in unstimulated (middle panels) and TNF-stimulated (right panels) PBMCs. Top and bottom panels represent two different healthy adult donors. Background fluorescence for phosphor-NF-κB is shown in the left panels (FMO). FMO, fluorescence minus one; IL, interleukin; PBMC, peripheral blood mononuclear cell; STAT5, signal transducer and activator of transcription 5; TNF, tumor necrosis factor.
(TIFF)

S1 Table Summary of all donors and measurements for ^14^C testing and downstream analysis. (XLSX)

S2 Table Results of all modeling from Figs 1–4. (XLSX)

S3 Table Results of robustness tests. (XLSX)

S4 Table Scenario testing for Fig 2 to define best fit scenarios for proportion of naive T cells that undergo turnover in the periphery. (XLSX)

S5 Table List of antibodies used for mass cytometry studies in Fig 6. (XLSX)

S1 Text Supplementary mathematical modeling information. (PDF)

S1 Data Raw data for Fig 5 Ki67 frequencies for different PBMC donors. PBMC, peripheral blood mononuclear cell.
(XLSX)

S2 Data Raw data for S4 Fig values for flow cytometry data on NF-κB p65 phosphorylation. NF-κB, nuclear factor κB.
(XLSX)

S3 Data Raw data for Fig 5E and 5F CFSE^low^ frequencies for different donors/conditions. CFSE, carboxyfluorescein succinimidyl ester.
(XLSX)

## Abbreviations

ΔAICc: differences in Akaike Information Criterion values
A.U.: arbitrary units
CFSE: carboxyfluorescein succinimidyl ester
CM: central memory
DREVI: conditional-density rescaled visualization
EM: effector memory
EMRA: terminal differentiated effector memory (CD45RA^+^)
ERK: extracellular signal–regulated kinase
FACS: fluorescence-activated cell sorting
FSC-A: forward scatter area
FSC-H: forward scatter height
IL: interleukin
n.s.: not significant
nd: no difference
NF-κB: nuclear factor κB
PBMC: peripheral blood mononuclear cell
PD-1: programmed cell death protein 1
PE: phycoerythrin
SSC-H: side scatter height
SSE: sum of squared errors
STAT5: signal transducer and activator of transcription 5
TNF: tumor necrosis factor
TREC: T-cell receptor excision circle
Tscm: stem cell memory T
t-SNE: t-stochastic neighborhood embedding
