# Supplementary figures and images for: Cell generation dynamics underlying naive T-cell homeostasis in adult humans
Source: PLoS Biol. 2019 Oct 29;17(10):e3000383. doi: 10.1371/journal.pbio.3000383 (PMC6818757; doi:10.1371/journal.pbio.3000383)

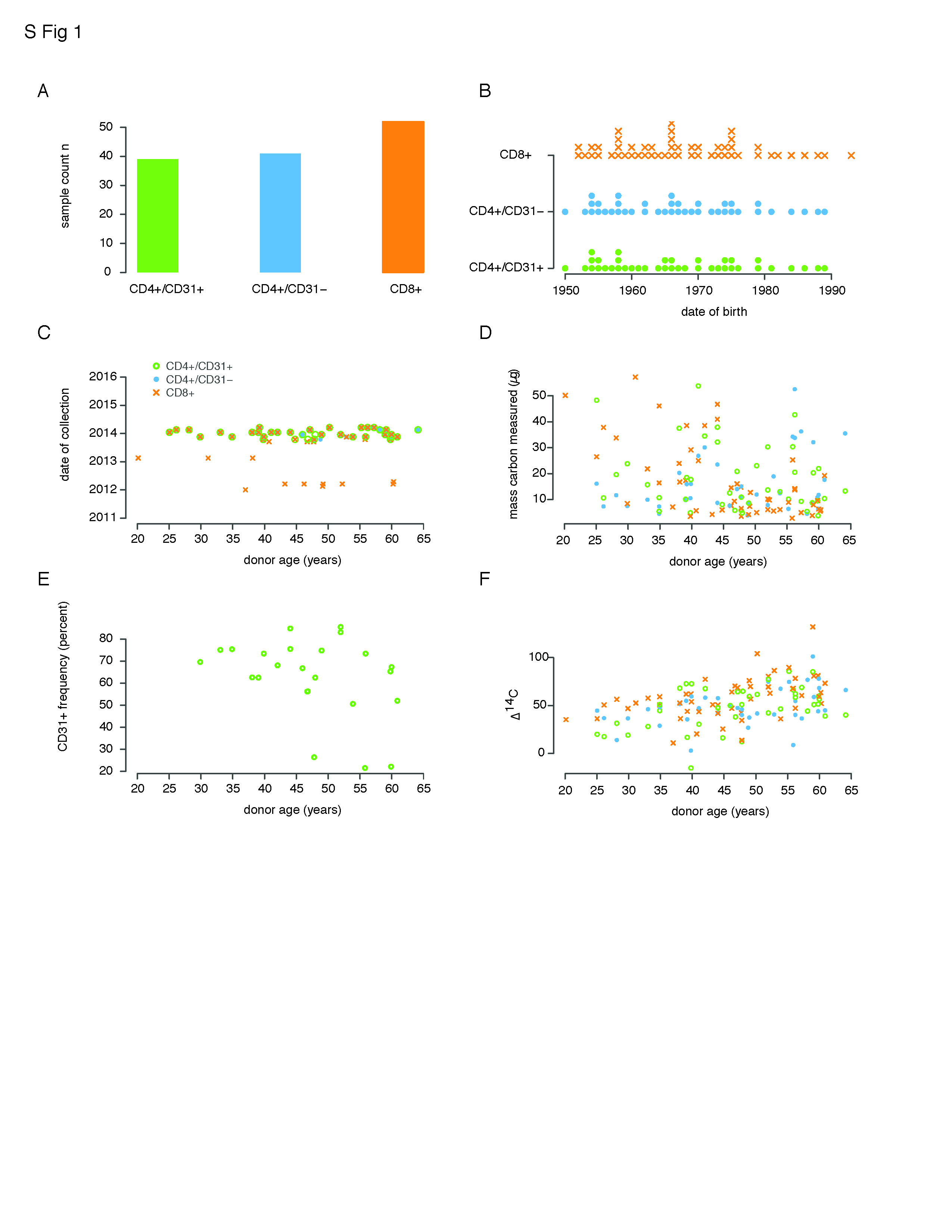

Supplement: S1 Fig — A complete overview is also provided in S1 Table. (A) Total number of samples included in 14C cell DNA age determination for Figs 1–4. (B) Distribution of samples relative to donor age (date of birth) for each sample set. (C) Collection date for each sample relative to donor age. Most samples were collected in 2014. (D) Carbon mass after isolation from purified DNA taken from each sample relative to donor age. (E) Frequency of CD31+ cells within the CD4+ naive T-cell fraction for all donors. (F) Summary of 14C measurements for all donors and cell types in study. (TIFF) [file pbio.3000383.s001.tiff]

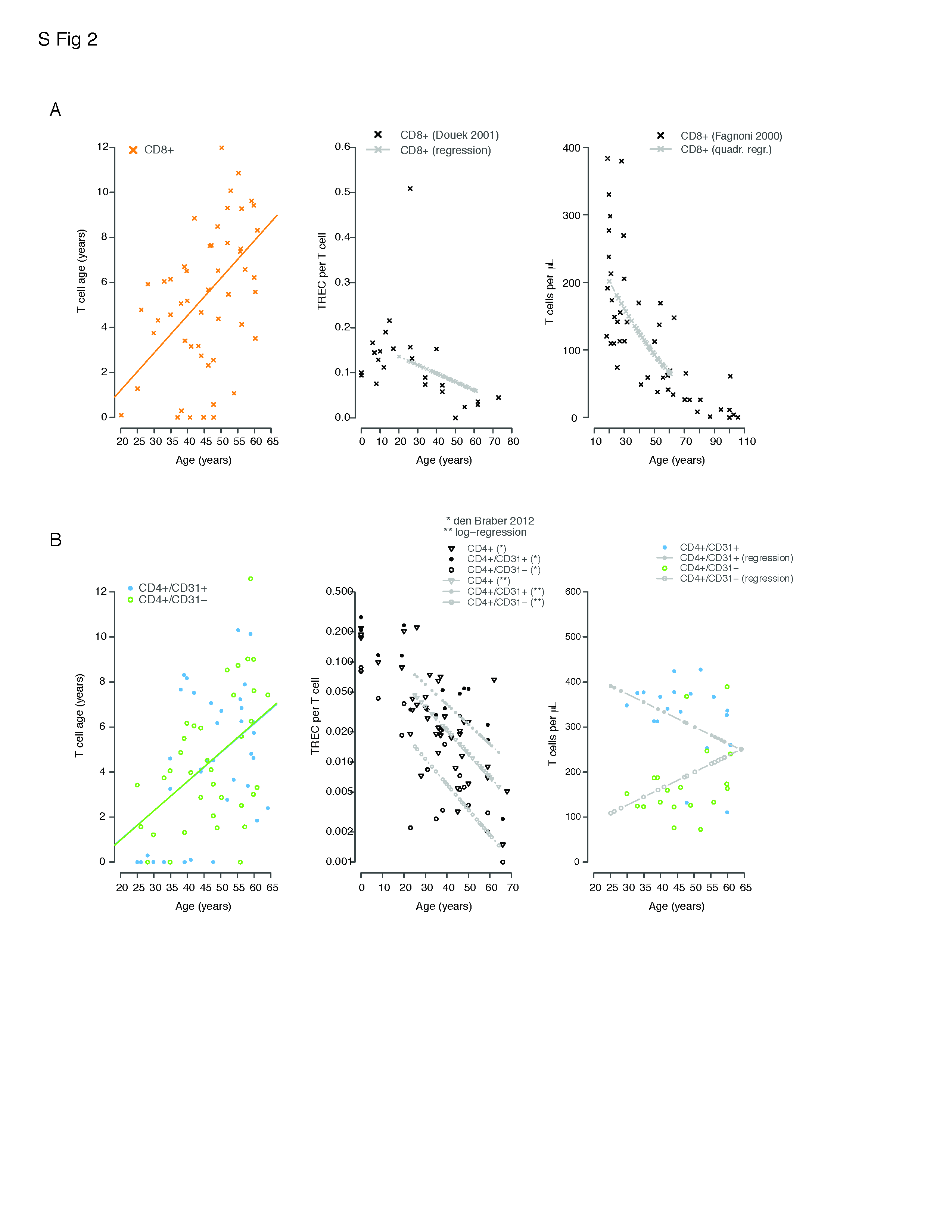

Supplement: S2 Fig — (A) CD8+ naive T-cell measurements for cell DNA age (based on this study), TREC content (taken from [23]), and cell numbers (taken from [13]). (B) CD4+ naive T-cell measurements for cell DNA age (based on this study), TREC content (taken from [8]), and cell numbers. Cell numbers were set to 500 cells/μl and defined in terms of CD31+ and CD31− fractions based on regressed data from S1E Fig. TREC, T-cell receptor excision circle. (TIFF) [file pbio.3000383.s002.tiff]

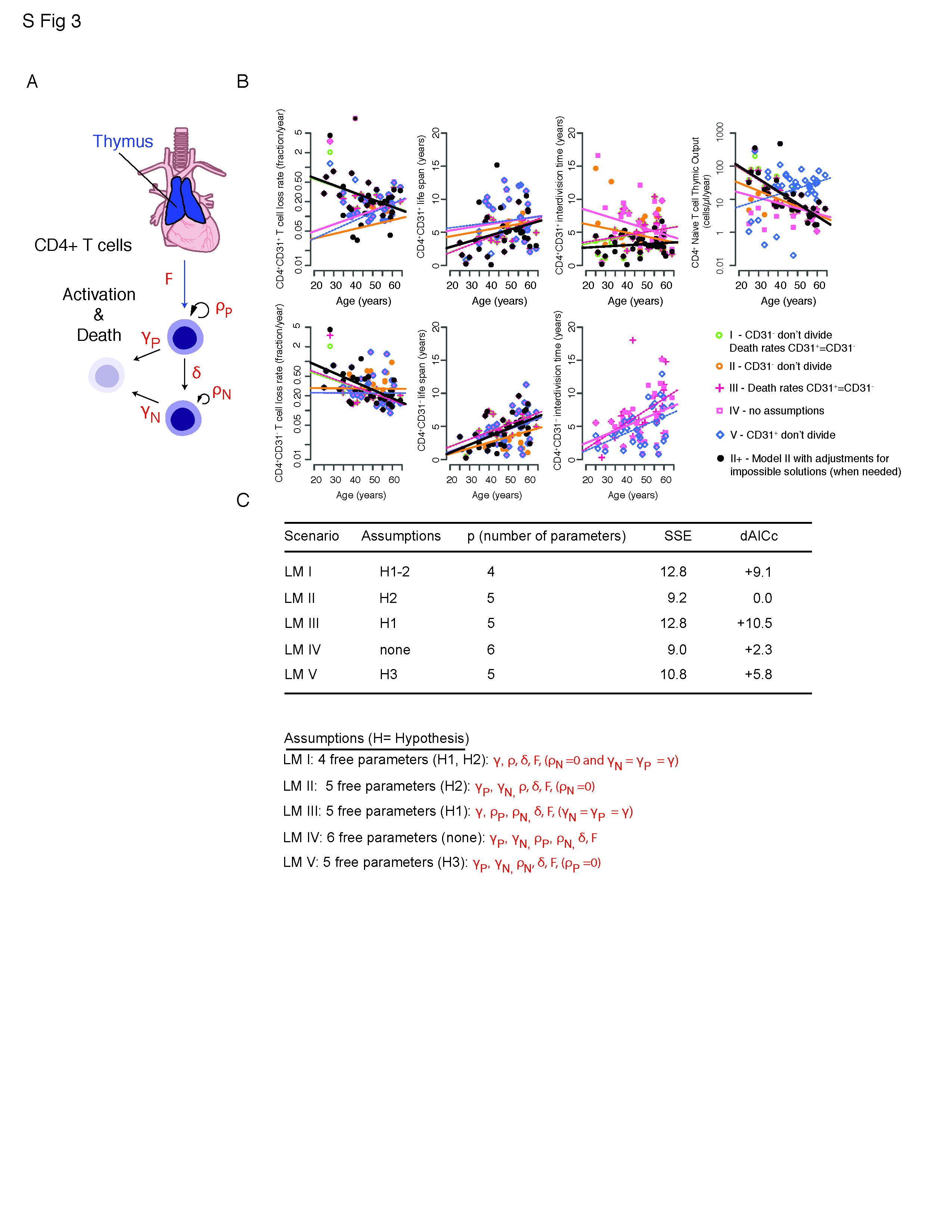

Supplement: S3 Fig — (A) Schematic of CD4+ naive T-cell production, proliferation, differentiation, and activation/death. (B) Representation of calculated dynamic values for each scenario tested. (C) Table indicating different features of each scenario (Linear Models I–V) and SSE and ΔAICc (“dAICc”) for each scenario. Hypotheses tested for each scenario are listed below. ΔAICc; differences in Akaike information criterion values; SSE, sum of squared errors. (TIFF) [file pbio.3000383.s003.tiff]

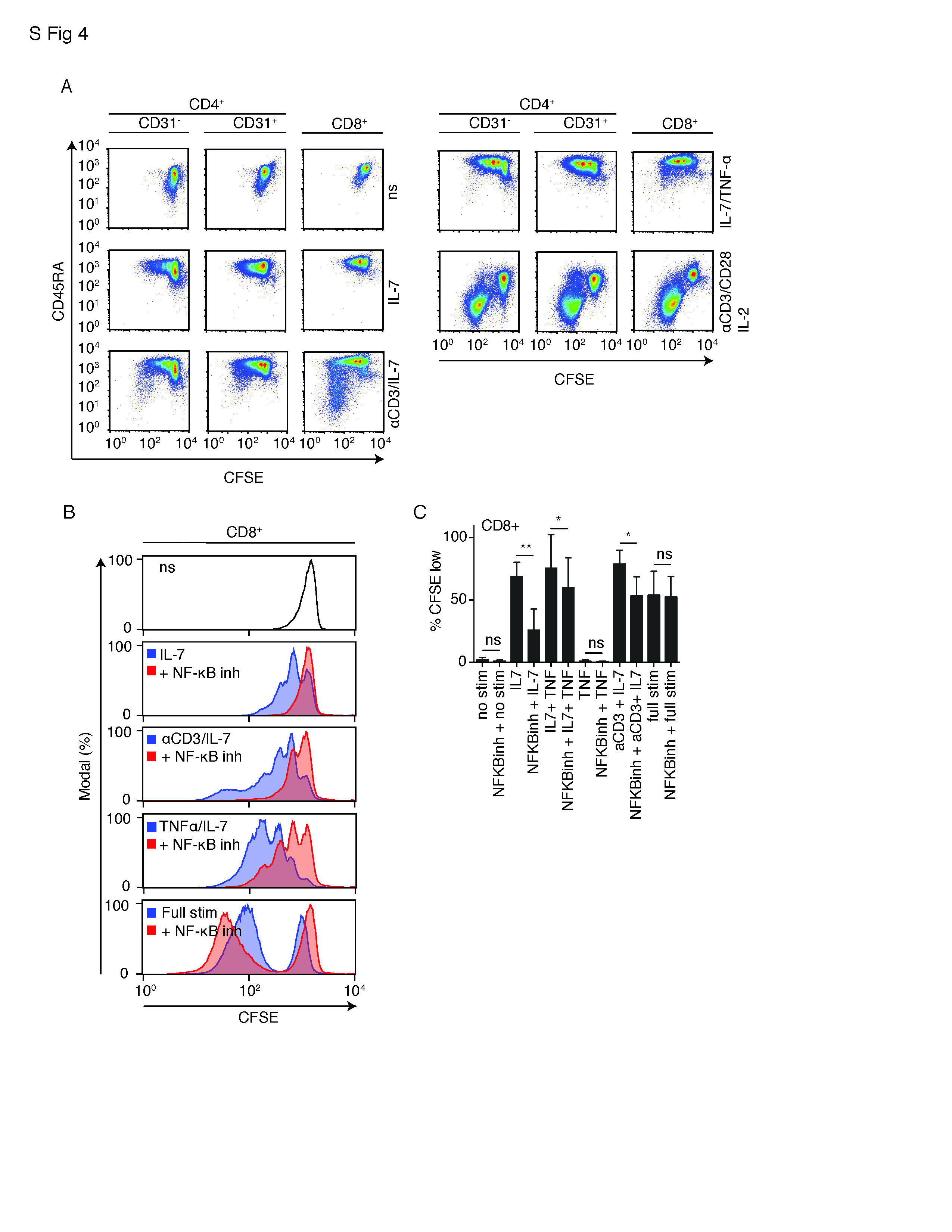

Supplement: S4 Fig — (A) CFSE dilution versus CD45RA expression for each stimulation condition. Minimal CD45RA down-regulation is observed with the exception αCD3 + IL-7 stimulation. Full stimulation (αCD3/αCD28 + IL-2) results in full activation of naive T cells. (B) CFSE dilution with different conditions for CD8+ naive T cells. Vehicle (DMSO—blue histograms) and treatment with NF-κB inhibitor (blue histogram) is shown. (C) Summary of four independent donors for CD8+ naive T cells. CFSE, carboxyfluorescein succinimidyl ester; IL, interleukin; NF-κB, nuclear factor κB. (TIFF) [file pbio.3000383.s004.tiff]

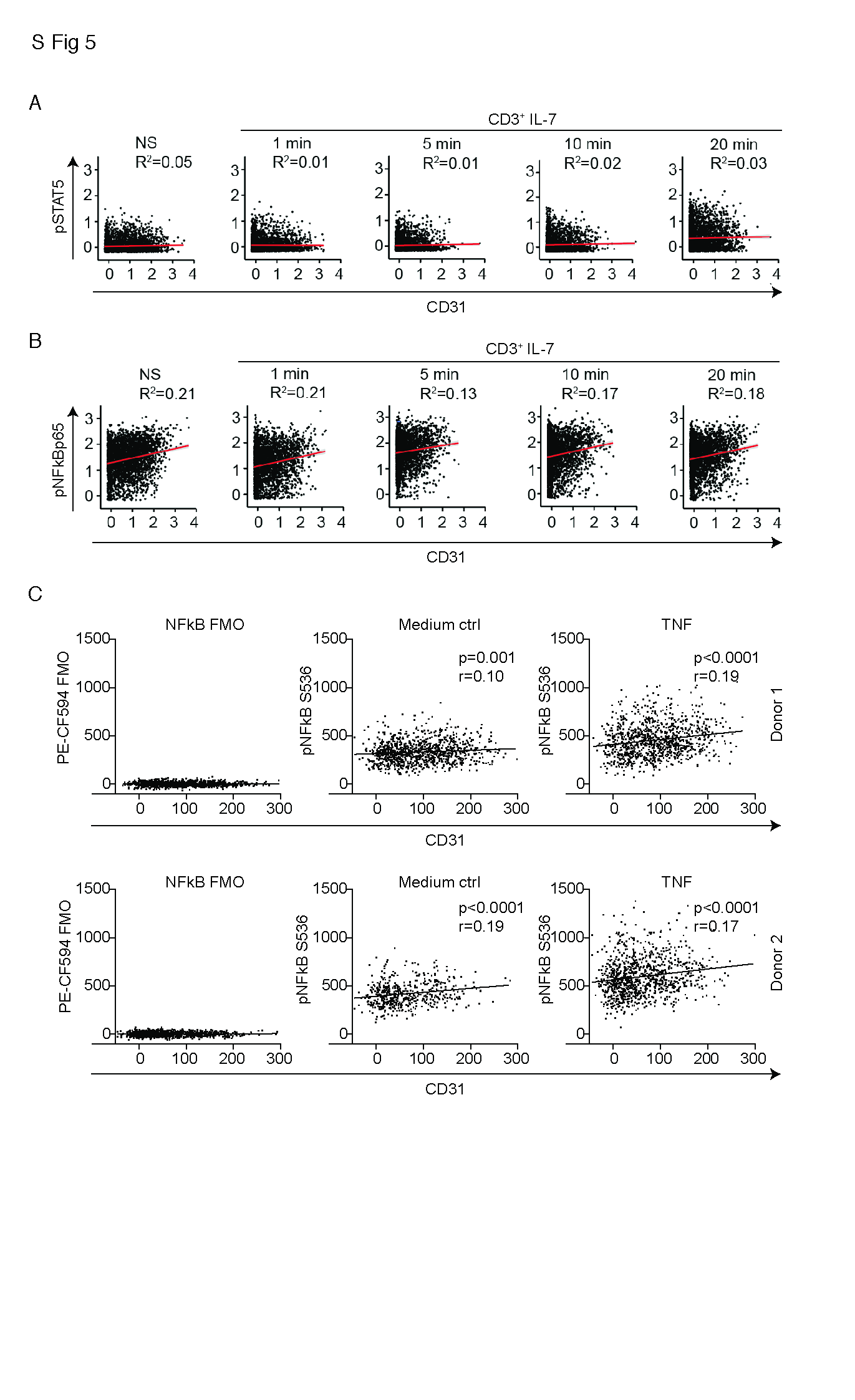

Supplement: S5 Fig — (A) Phosphorylation of STAT5 in unstimulated (“NS”) and in αCD3 + IL-7 (10 ng/mL) stimulated PBMCs. CD4+ naive T cells are identified by gating on lineage negative, CD3+CD4+CD45RA+CD27+CCR7+ and Pearson’s correlations are depicted for phosphor-STAT5 (y-axis) versus CD31 expression (x-axis). Values for different time points post-stimulation are shown (B) Phosphor-NF-κB (RelA/p65) versus CD31 expression on the same populations as in (A). (C) Confirmation of CyTOF results using flow cytometry to identify naive T cells (gate: Live, Lineage Negative, CD3+CD4+CD45RA+CCR7+) and monitoring phosphor-NF-κB (RelA/p65) (y-axis) versus CD31 expression (x-axis) in unstimulated (middle panels) and TNF-stimulated (right panels) PBMCs. Top and bottom panels represent two different healthy adult donors. Background fluorescence for phosphor-NF-κB is shown in the left panels (FMO). FMO, fluorescence minus one; IL, interleukin; PBMC, peripheral blood mononuclear cell; STAT5, signal transducer and activator of transcription 5; TNF, tumor necrosis factor. (TIFF) [file pbio.3000383.s005.tiff]
