# Supplementary material for: Cell generation dynamics underlying naive T-cell homeostasis in adult humans
Source: PLoS Biol. 2019 Oct 29;17(10):e3000383. doi: 10.1371/journal.pbio.3000383 (PMC6818757; doi:10.1371/journal.pbio.3000383)
Supplement: S1 Text — (PDF) [file pbio.3000383.s011.pdf]

Supplemental Mathematical Methods

Cell generation dynamics underlying naive T cell homeostasis in adult humans

Jeff E. Mold, Pedro Réu, Axel Olin, Samuel Bernard, Jakob Michaëlsson, Sanket Rane, Andrew Yates, Azadeh Khosravi, Mehran Salehpour, Göran Possnert, Petter Brodin, and Jonas Frisén

---

General modeling strategies and methods

The model for CD4$^+$ and CD8$^+$ naive T cell dynamics is a generic birth/death/immigration model. Cells can be added through an external source, they can divide or be removed permanently from the naive T cell pool, through death or activation. The birth/death/immigration model covers a wide class of actual models, for which particular implementations can vary considerably in methods, whether stochastic or deterministic processes are considered, and in complexity, from simple linear one-step processes to nonlinear processes in heterogeneous populations. To reconstruct the T cell dynamics, we used two steps.
In the first step, we estimate the average age of each cell types in individual donors. In the second step, we use average age of cells to infer T cell dynamics.

1. The age of naive T cells were estimated using linear **age-structured equations** (ASE) Based on ^{14}C data from donors within defined age groups, we could determine the average age of the cells in each individual donor. The models and methods are described in Section 1 ASE.

2. To infer naive T cell dynamics, we tested a family of **linear models** (LM) for cell renewal in individual donors to determine loss, peripheral proliferation, and thymic production rates and, in the case of CD4$^+$ T cells, the probability for T cells to lose the CD31 marker. To do that, we made a steady-state assumption, i.e. the dynamical states and the kinetic parameters remain constant. The models and methods are described in Section 2 LM.

When possible, the corrected **Akaike Information Criterion (AICc)** was used to compare different scenarios. If the sum of square of the residuals between the model prediction and the data is denoted SSE, the number of data points is n, and the number of parameters p is $p = K - 1$, the AICc for the least-square problem is (Burnham and Anderson, 2002)

$$AIC_c = n \log\Big(\frac{SSE}{n}\Big) + 2K + \frac{2K(K+1)}{n-K-1}.$$

Comparison between models is reported as the difference in AICc, ΔAICc. Lower AICc means a more appropriate model.

The hierarchy of models is as follows. Stars indicate the selected model. Arrows show the dependence of the models. LM models used cell age estimates from ASE models.

```
  Model             Description
  -----------       -------------------------------------------
  ASE               Age-Structured Equations
.--*ASE-A           one-population constant turnover rate
|   ASE-2POPA       two-population constant turnover rate
|
`> LM               Linear Models
     LMI            no CD31- proliferation same loss rates
    *LMII           no CD31- proliferation different loss rates
     LMII+          LMII with positive solutions
     LMIII          proliferation same loss rates
     LMIV           proliferation different loss rates
     LMV            no CD31+ proliferation
```

Modeling naive T cell turnover in human

1 ASE - Estimating average cell age with an age-structured birth-and-death process from ^{14}C data

Keeping in mind that the atmospheric ^{14}C data provide information on the average age of the DNA, we want to track the average age of each cell, as defined by the age of the DNA.

Once synthesized, DNA is very stable. ^{14}C content in DNA reflects the time at which it was synthesized. A single naive T cell may have undergone divisions at different time points in the past, and different DNA strands will have different ages. The **average age** of a cell is defined as the average age age of all its DNA strands (92 strands: 2 strands per chromosome x 46 chromosomes). At division, daughter cells inherit half of the mother's old DNA, and half freshly synthesized DNA. We assume that old DNA is passed symmetrically to the daughter cells.

In an individual aged t years, we denote by $N(t)$ the total cell number. To take into account the age of the cells, we break down the population $N(t)$ into a continuum of age bins. The population structured in average cell age a. The new dynamical variable $n(t, a)$ is the density of cells of age a at time t. The cell

number and the cell density are related in the following way

$$N(t) = \int_0^t n(t,a)da.$$

Atmospheric ^{14}C levels have been measured in the northern and southern hemispheres over the past decades, at distinct time points y_i, expressed in calendar years, $i = 1, ..., N_y$. In practice, we use a linear interpolation $K(y)$ of y_i. The ^{14}C half-life of 5568 years plays only a minor role for contemporary carbon dating. The ^{14}C levels in a cell population measured at calendar year d (the collection date) are found by averaging the atmospheric profile K against the normalised density $n(t,a)$,

$$c = \int_0^t \frac{n(a,t)}{N(t)} K(d-a)da.$$

A first exploration of the data confirmed that naive T cells were much younger than the donors. Therefore, complex, lifelong changes in cell renewal dynamics could be ignored when estimating average ages of the cells. Short-lived cells have a short "memory" of past events, which makes inference about long-term dynamics impossible. To describe the age-structured model completely, we make a set of assumptions

1. Constant loss rate r (unit: per year).
2. At time $t_0 = 0$ (birth of the individual), all N_0 cells are aged 0 year.
3. Constant birth rate $\beta = r$. This assumption means that cell number remains constant.

The age-structured model can be implemented as a linear PDE with a linear loss rate. Let n be the density of cells with average age a in an individual of age t. In absence of any event, the average age of all cells increases by one year every year. The PDE is

$$\frac{\partial n}{\partial t}(a,t) + \frac{\partial n}{\partial a}(a,t) = -rn(a,t) \tag{ASE}$$

on $a \in]0,+\infty]$, for $t > 0$. We specify the initial boundary value problem with initial conditions $t = 0$ for $a \in]0,+\infty]$ and the boundary conditions for $t \geq 0$,

$$n(a,0) = 0, \text{ for } a \in]0,+\infty], \tag{IC}$$

$$n(0,t) = \begin{cases} N_0\delta(a) & \text{if } t = 0, \\ \beta & \text{if } t > 0. \end{cases} \tag{BC}$$

The loss rate in the right-hand-side of equation (ASE) comes from assumption 1. The Dirac delta distribution δ in equation (BC) comes from assumption 2. The boundary condition β in equation (BC) comes from assumption 3. In this model, the condition $n(t,0) = \beta$ means that new cells have an average age of 0

years. This is a reasonable approximation of the cell has been produced in the thymus, where it has undergone several rounds of divisions. Peripheral division produces daughter cells that have intermediate average ages. From the point of view of DNA, the thymic production and peripheral division preserve the average age. A new set of DNA of age 0 years has been added, even though the density $n(t, a)$ would not be affected. Since DNA from several million cells is pooled for the ^{14}C samples, there is no way, and no need, to distinguish between sources of cells at this stage of the modeling.

Data All data are available in `S Table 1`, table `radiocarbon_samples`. ^{14}C levels in samples of CD4$^+$/CD31$^+$ cells, CD4$^+$/CD31$^-$ cells, and CD8$^+$ cells from healthy donors (column `Delta14C`); dates of collection of the samples (column `Date of Collection`); dates of birth of the donors (column `Year of Birth`).

Fitting strategy. For each T cell subset (CD4$^+$/CD31$^+$, CD4$^+$/CD31$^-$, CD8$^+$), the loss rates r were estimated using a constant, homogeneous turnover scenario (scenario A) or a constant turnover rate restricted to a fraction of the cells (scenario 2POPA). The parameters were estimated by a nonlinear least-square on each of the subset (CD4$^+$/CD31$^+$, n=46; CD4$^+$/CD31$^-$, n=44; CD8$^+$, n=52).

Outcome. The two scenarios predict similar turnover rates in all cell types. Scenario 2POPA predicts that 100% of the cells are subject to renewal. This means that there is no long-lived cell pool (Table 1).

Table 1: Scenarios for average cell age based on the **age-structured model**, for CD4$^+$ and CD8$^+$ cells. A: all cell turn over at a constant rate r (*column A* r). 2POPA: only a fraction f (*column 2POPA* f) of cells turn over at a constant rate r (*column 2POPA* r). The remaining $(1 - f)$ fraction is long-lived and does not turn over. ΔAICc is the differential AICc between Scenarios 2POPA and A, and a positive value indicates that Scenario A should be preferred. In all cell types, the estimated fraction of cells that turn over is very close to 1.0, indicating that long-lived cells form a negligible part of the population. This conclusion is supported by the fact that the sum-of-square of the errors (SSE) for Scenario A and 2POPA are the same, and that the values of ΔAICc are positive.

Type	A r	A SSE	2POPA r	2POPA f	2POPA SSE	ΔAICc
CD31$^+$	2.674e-01	6.812e+06	2.489e-01	1.004e+00	6.812e+06	2.292
CD31$^-$	2.523e-01	3.932e+06	2.461e-01	1.003e+00	3.932e+06	2.307
CD8$^+$	2.128e-01	4.036e+06	2.129e-01	9.998e-01	4.036e+06	2.255
CD4$^+$	2.606e-01	1.075e+07	2.479e-01	1.004e+00	1.075e+07	2.140

Given that all cells in each subset are subject to renewal, loss rates could be estimated for each individual ^{14}C sample. The average age $\langle a \rangle$ of cells was

computed based on the loss rate estimate r and the age of the donor t using the relationship

$$\langle a \rangle = \frac{1 - \exp(-\gamma t)}{\gamma}.$$

Average cell ages are reported in `S Table 3` (table `average_cell_ages`, column `average cell DNA age`).

2 LM - Estimating individual kinetic parameter sets with a linear model

All naive T cells are assumed to have the following characteristics: CD31 marker expression, average cell DNA age, and TREC content. Naive T cells can be produced in the thymus. Naive T cells can divide or be lost in the periphery. Cell loss includes removal through death, activation, migration, etc.

Cell dynamics

- Thymic production. $CD31^+$ cells are produced in the thymus and released in the periphery at a rate F.
- Peripheral proliferation. $CD31^+$ cells can proliferate at a rate ρ_P, and are lost at a rate γ_P.
- At each division, $CD31^+$ cells lose their marker with a probability δ.
- $CD31^-$ cells die at a rate γ_P and proliferate at a rate ρ_N.

Average age

- $CD31^+$ cells produced in the thymus have an average DNA aged 0 year.
- When a cell divides, average cell age is divided by two.

TREC (T-cell receptor excision circles)

- TRECs are generated in the thymus only. The TREC content of cells exiting the thymus is T_0 Early observations [Douek et al 1998, Nature] noted that cells undergo 3-4 divisions between TCRD and TCRA rearrangement, i.e. after the TREC product from TCRD deletion has been created, suggesting a $1/2^3$=0.125 dilution in TREC content.
  TREC content in mature thymocytes and in CD4+CD31+ naive T cells in the cord blood of neonates is roughly 0.25 [den Braber et al (2012)], we have therefore used the value of 0.25.
- During cell division, TRECs are passed to daughter cells with equal probability.

- TRECs are lost only through cell loss.

From these modeling assumptions, we can construct an ODE model for naive T cell dynamics,

$$\frac{dP}{dt} = F - \gamma_P P + \rho_P(1 - 2\delta)P, \tag{1}$$

$$\frac{dN}{dt} = -\gamma_N N + 2\rho_P \delta P + \rho_N N, \tag{2}$$

$$\frac{dT_P}{dt} = T_0 F - \gamma_P T_P - \rho_P \delta T_P, \tag{3}$$

$$\frac{dT_N}{dt} = -\gamma_N T_N + \rho_P \delta T_P, \tag{4}$$

$$\frac{dA_P}{dt} = 1 - \gamma_P A_P - \rho_P \delta A_P - \frac{\dot{P}}{P} A_P, \tag{5}$$

$$\frac{dA_N}{dt} = 1 - \gamma_N A_N + \rho_P \delta \frac{P}{N} A_P - \frac{\dot{N}}{N} A_N, \tag{6}$$

where $\dot{P} = \frac{dP}{dt}$ and $\dot{N} = \frac{dN}{dt}$.

The dynamical variables are: P the total CD31$^+$ T cell density; N the total CD31$^-$ T cell density; T_P the total CD31$^+$ T cell TREC content; T_N the total CD31$^-$ T cell TREC content; A_P the average CD31$^+$ T cell age; A_N the average CD31$^-$ T cell age. Cell densities are normalized with respect to the initial cell number; TREC content in cells exiting the thymus is $T_0 = 0.25$ TREC per cell; and the average age is in years. This model is similar to the ones proposed by Bains et al (2009), with the difference that the average cell age is taken into account.

Equations (1-2) describe the density of naive CD31$^+$ and CD31$^-$ T cells respectively. Naive CD31$^+$ T cells are produced at a rate F from the thymus; they are lost at a rate γ_P; they divide at rate ρ_P. There is a probability δ for the daughter cells to lose their CD31 marker, so that the net rate within the CD31$^+$ compartment is $2\rho_P(1 - \delta) - \rho_P = \rho_P(1 - 2\delta)$. The first term counts the 2 daughter cells and the second term counts the leaving mother cell dividing. Likewise, naive CD31$^-$ T cells are lost at a rate γ_N; they are produced at a rate $2\rho_P\delta$ and proliferate at rate ρ_N.

Equations (3-4) describe the total TREC content in naive CD31$^+$ and CD31$^-$ T cells respectively. Cell division does not change the total TREC content. Naive CD31$^+$ T cell TRECs are produced in the thymus at rate T_0F; they are lost at rate γ_P and are transferred to naive CD31$^-$ T cells at rate $\rho_P\delta$. Naive CD31$^-$ T cell TRECs are lost at rate γ_N and are produced at rate $\rho_P\delta T_P$.

Equations (5-6) for average T cell ages can be obtained by writing down the corresponding age-structured equations and integrating them with respect to

donor age. The equation for the age-density $p(t,a)$ of naive CD31$^+$ T cells reads

$$\frac{\partial p}{\partial t}(t,a) + \frac{\partial p}{\partial a}(t,a) = -\gamma_P p(t,a) + 2\rho_P(1-\delta)2p(t,2a) - \rho_P p(t,a). \tag{7}$$

The factor 2 in front of it accounts for the conservation of mass. Daughter cells with average ages between $[a, a+da]$ have parental cells with average ages included in an interval twice as large $[2a, 2a+2da]$, so as cells divide the age density get more concentrated by a factor 2.

The equation for the age-density $n(t,a)$ of naive CD31$^-$ T cells reads

$$\begin{aligned}\frac{\partial n}{\partial t}(t,a) + \frac{\partial n}{\partial a}(t,a) = -\gamma_N n(t,a) &+ 2\rho_P\delta 2p(t,2a) \\ &+ 2\rho_N 2n(t,2a) - \rho_N n(t,a).\end{aligned} \tag{8}$$

Given the cell densities $p(t,a)$ and $n(t,a)$, the cell population average ages A_P and A_N are

$$A_P(t) = \frac{\int_0^\infty ap(t,a)da}{P(t)}, \tag{9}$$

$$A_N(t) = \frac{\int_0^\infty an(t,a)da}{N(t)}. \tag{10}$$

Note that the total cell densities $P(t)$ and $N(t)$, given in equations (1-2), also satisfy the relations

$$P(t) = \int_0^\infty p(t,a)da, \tag{11}$$

$$N(t) = \int_0^\infty n(t,a)da. \tag{12}$$

Integrating the CD31$^+$ T cell age-density equation with $\int_0^\infty da\, a\cdot$ yields

$$\begin{aligned}\frac{d}{dt}\int_0^\infty ap(t,a)da + \int_0^\infty a\frac{\partial p}{\partial a}(t,a)da = &-\gamma_P\int_0^\infty ap(t,a)da \\ &+ 2\rho_P(1-\delta)2\int_0^\infty ap(t,2a)da \\ &- \rho_P\int_0^\infty ap(t,a)da.\end{aligned}$$

The term

$$\frac{d}{dt}\int_0^\infty ap(t,a)da = \frac{d}{dt}(A_P P) = P\frac{d}{dt}A_P + A_P\frac{d}{dt}P$$

The term

$$\int_0^\infty a\frac{\partial p}{\partial a}(t,a)da = ap(t,a)|_0^\infty - \int_0^\infty p(t,a)da = 0 - P.$$

The term $\int ap(t,a)da = A_P(t)P(t)$ and the term $\int ap(t,2a)da = A_P(t)P(t)/4$. Gathering all the terms, we obtain the ordinary differential equation

$$P\frac{d}{dt}A_P + A_P\frac{d}{dt}P - P = -\gamma_P A_P P + \rho_P(1-\delta)A_P P - \rho_P A_P P.$$

Equation (5) is obtained by dividing by $P(t)$. Equation (6) is obtained in a similar way by integrating the CD31$^-$ T cell age-density equation.

CD4$^+$ individual parameter estimates

Implementation of the LM model The LM model corresponds to the steady-states of the ODE system with constant kinetic parameters where all derivatives are set to 0.

CD4$^+$ data and fitting strategy for the linear model. Individual data we use come from a subset of 36 donors for which average cell ages in both CD4$^+$/CD31$^+$ and CD4$^+$/CD31$^-$ fractions could be estimated:

- average cell age in CD4$^+$/CD31$^+$ (A_P) and CD4$^+$/CD31$^-$ (A_N) T cells obtained with the age-structured model (ASE).
- CD4$^+$/CD31$^+$ T cell frequency among circulating CD4$^+$ R. The frequency was available for a subset of the donors only. For the remaining donors, we performed a linear regression to estimate the expected frequency.
- TREC content in the CD4$^+$/CD31$^+$ (T_P) and CD4$^+$/CD31$^-$ (T_N) T cell subsets, or in all CD4$^+$ T cells (T). TREC content was not available for this study and was inferred by log-linear regression from published data [den Braber et al (2012)].
- Total CD4$^+$ cell number (C). Total circulating CD4$^+$ T cell number was set to 500 cells/μL blood (Kilpatrick et al. 2008).

The full dataset consists of 6 observables (average CD31$^+$ cell age, average CD31$^-$ cell age, CD31$^+$ frequency, Total CD31$^+$ TREC content, Total CD31$^-$ TREC content, Total cell number) for each of donor. Average cell DNA age in CD31$^+$ and CD31$^-$ naive T cells are found in `S3_Table`, table `CD4_all_models_parameter_identification`. TREC contents are found in `S1_Table`, tables `trec_CD4_total` and `trec_CD4_posneg`. CD31$^+$ naive T cell frequencies are found in `S1_Table` , table `radiocarbon_samples`. Data are illustrated in S Fig 2B.

The goal of the LM model is to estimate kinetic parameters in each donor. This is done by assuming that the dynamics of the T cells are at steady state, i.e. that

the observables and the parameters are constant over the characteristic time for T cell turnover, i.e. around 5 years. With the steady state assumption, a nonlinear system of equations can be obtained from the ODE system by setting all derivatives to zero. This leads to a system of 6 nonlinear algebraic equations, where six dynamical variables $(P, N, T_P, T_N, A_P, A_N)$ can be expressed in terms of the 6 observables. The parameter estimation problem can then be reduced to the problem of solving this system of 6 nonlinear equations In general it is difficult to determine if there is a solution, and if so, whether it is unique. Fortunately, this system is almost linear in the model parameters, with a single nonlinear interaction in the form of the term $\rho_P\delta$. The parameter δ only appears within that term, so $\rho_P\delta$ can be considered as a single parameter. This way the nonlinear problem reduces to a linear system, for which we know exactly when solutions exists and when they are unique. Briefly, the linear problem is in the form $Mx = b$, where M is a matrix with 6 rows, one for each equation, and p columns, one for each parameter to identify. The variable x is an unknown parameter vector of size p, and b is a constant vector of size 6. The full model for naive $CD4^+$ T cells (six equations and six unknowns) is

$$\begin{bmatrix} -P & 0 & -2P & P & 0 & 1 \\ 0 & -N & 2P & 0 & N & 0 \\ -T_P/T_0 & 0 & -T_P/T_0 & 0 & 0 & 1 \\ 0 & -T_N & T_P & 0 & 0 & 0 \\ A_P & 0 & A_P & 0 & 0 & 0 \\ 0 & -A_N & \frac{A_P P}{N} & 0 & 0 & 0 \end{bmatrix} \begin{bmatrix} \gamma_P \\ \gamma_N \\ \rho_P\delta \\ \rho_P \\ \rho_N \\ F \end{bmatrix} = \begin{bmatrix} 0 \\ 0 \\ 0 \\ 0 \\ 1 \\ -1 \end{bmatrix}. \tag{13}$$

The number of parameters to estimate, p, can be smaller than 6 if the model is simplified. If $p = 6$, the problem has a unique solution if and only if M is full rank (i.e. is invertible). If M is not full rank, the problem is under-determined and not all parameters can be identified. If $p < 6$, and M is full rank, the problem is over-determined (too many constraints) and it is treated, as is usually done, as a least-square problem (linear regression). The least-square problem has a unique solution if rank $M \geq p$.

The total number of free parameters that can be estimated with the **linear system** is 6: γ_P, γ_N, ρ_P, ρ_N, δ, F. Biological hypotheses can be explored by setting specific values to one or more of the parameters and comparing how good parameter estimates are with the AICc. We tested three biological hypotheses: (H1) removal rates are the same in the $CD31^+$ and the $CD31^-$ fraction of the $CD4^+$ population, (H2) the proliferation rate of the $CD31^-$ fraction is zero, and (H3) the proliferation rate of the $CD31^+$ fraction is zero. This leads to 5 combinations of biological scenarios.

I. 4 free parameters (H1,H2): $\gamma, \rho, \delta, F; \rho_N = 0, \gamma_N = \gamma_P = \gamma$.

II. 5 free parameters (H2): $\gamma_P, \gamma_N, \rho, \delta, F; \rho_N = 0$.

III. 5 free parameters (H1): $\gamma, \rho_P, \rho_N, \delta, F; \gamma_N = \gamma_P = \gamma$.

IV. 6 free parameters (full model, no hypothesis): $\gamma_P, \gamma_N, \rho_P, \rho_N, \delta, F$.

V. 5 free parameters (H3): $\gamma_P, \gamma_N, \rho_N, \delta, F$; $\rho_P = 0$.

Outcome. For each scenario, and for each CD4$^+$ cell samples, we solved the linear least-square problem using the function `lsqnonneg` from Matlab (Mathworks, Natick, MA), which computes the least-square non-negative solution. We summed up the square error of individual fits to get an SSE (sum of square errors). For each model, the AICc was calculated based on SSE, (Table 2).

Table 2: LM models. Five models for individual CD4$^+$ cell kinetic parameter estimates are compared. LMI: $p = 4$ parameters, CD31$^+$ and CD31$^-$ fractions have the same removal rates (H1), and CD31$^-$ cells do not divide (H2). LMII: $p = 5$ parameters, CD31$^-$ cells do not divide (H2). LMIII: removal rates are the same (H1). LMIV: full model, no hypothesis. LMV: no proliferation in the CD31$^+$ fraction (H3). Lower ΔAICc correspond to better models. LMII was the best scenario, followed by LMIV.

Scenario	assumptions	p (number of parameters)	SSE	ΔAICc
LMI	H1-2	4	12.8	+9.1
LMII	H2	5	9.2	0.0
LMIII	H1	5	12.8	+10.5
LMIV	none	6	9.0	+2.3
LMV	H3	5	10.8	+5.8

LMII is the best one according to AICc, so we decided to use it as the basis for for further modeling. The linear problem for LMII is

$$\begin{bmatrix} -P & 0 & -2P & P & 1 \\ 0 & -N & 2P & 0 & 0 \\ -T_P/T_0 & 0 & -T_P/T_0 & 0 & 1 \\ 0 & -T_N & T_P & 0 & 0 \\ A_P & 0 & A_P & 0 & 0 \\ 0 & -A_N & \frac{A_P P}{N} & 0 & 0 \end{bmatrix} \begin{bmatrix} \gamma_P \\ \gamma_N \\ \rho\delta \\ \rho \\ F \end{bmatrix} = \begin{bmatrix} 0 \\ 0 \\ 0 \\ 0 \\ 1 \\ -1 \end{bmatrix}. \quad (14)$$

Although model II was selected, the solutions computed with `lsqnonneg` led to several zero parameter values for the death rates and differentiation rates (`lsqnonneg` does not guarantee positive solutions). These solutions were suboptimal because it led to either very old CD31$^-$ cells, or very few of them. To obtain strictly positive parameter estimates, we used the following alternative parameter identification strategies. `S Table 3`, table `CD4_LMIIpositive_parameter_identification`, column `Type of parameter identification` shows the strategy used for each donor.

- If $A_P > 0$, and all estimated parameters greater than 0, then the least-square solution to LMII was accepted. This is solution *IIa* obtained by solving equation (13).
- If $A_P = 0$, then the matrix A is rank-deficient and not all 5 parameters can be estimated. This is solution *IIb*, detailed below.
- If parameters values obtained by non-negative least-square were zero, then a non-optimal positive solution was selected. This is solution *IIc*, detailed below.

IIb. When $A_P = 0$, the matrix M has rank 4 (the fifth row vanishes and rows 2, 4 and 6 are co-linear), and not all 5 parameters can be estimated. In this case, the loss rate γ_P was set to the maximum between $\rho\delta$ and γ_N to ensure positive solutions, and the other parameters were solved exactly,

$$\gamma_N = \frac{1}{A_N}, \tag{15}$$

$$\rho\delta = \frac{T_N(1-R)}{T_P R A_N}, \tag{16}$$

$$\gamma_P = \max\{\rho\delta, \gamma_N\}, \tag{17}$$

$$F = \big(\gamma_P - \rho\delta\big) T_P / T_0 C R, \tag{18}$$

$$\rho = \frac{\gamma_P R - F/C + 2\delta\rho R}{R}. \tag{19}$$

IIc. When $A_N = 0$ or when the non-negative least-square estimates $\delta = 0$ or $\gamma_P = 0$, the parameters were set to

$$\gamma_P = \gamma_N, \tag{20}$$

$$\gamma_P = \frac{1}{A_P R + A_N(1-R)}, \tag{21}$$

$$F = \gamma_P C T / T_0, \tag{22}$$

$$\delta\rho = \frac{\gamma_P(1-R)}{2R}, \tag{23}$$

$$\rho = \gamma + 2\delta\rho - \frac{F}{CR}. \tag{24}$$

Together these assumptions define **LMII+**, which was used as the reference model for further statistical analysis.

In LMII, $CD31^+$ and $CD31^-$ cells have different death rates. There was however no difference between identified death rates ($CD4^+/CD31^+$ and $CD4^+/CD31^-$ cells; p-value = 0.16, Welch two-sample t-test).

Note on the importance of cell age Information on average cell age is essential for parameter identification. When cell age is lacking, the linear

problem reduces, in the best case, to

$$\begin{bmatrix} -P & -2P & P \\ -N & 2P & 0 \\ T_P/T_0 & T_P/T_0 & 0 \\ -T_N & T_P & 0 \end{bmatrix} \begin{bmatrix} \gamma \\ \rho\delta \\ \rho \end{bmatrix} = \begin{bmatrix} F \\ 0 \\ F \\ 0 \end{bmatrix}. \tag{25}$$

Thymic production (or any another parameter) has to go to the right-hand-side of the system, otherwise the only solution would be an all zero solution. Once thymic production is fixed, the linear problem becomes invertible, and there is a unique solution for each value of the thymic production F. Therefore, unique parameter identification depends on independent knowledge of F. Data on average age of cells provided this information.

CD8$^+$ per subject parameter estimates

CD8$^+$ data and fitting strategy.

The LM approach for CD8$^+$ T cell parameter identification followed a strategy similar to the one used for CD4$^+$ T cells. Because most of the CD8$^+$ T cells are also positive for the CD31 marker, the steady state equations were slightly modified. In particular, the average cell age and TREC content were available for the whole CD8$^+$ population only. The 4 observables were

- Average cell age A estimated with the age-structured model (ASE). Data are found in `S Table 3`, table ‘`CD8_parameter_identification`.
- TREC content T. TREC content in CD8$^+$ T cells was not available for this study, but was inferred by linear regression from published data [Douek et al. (2001)]. Data are found in `S Table 1`, table `trec_CD8`.
- CD31 frequency R. The CD31 frequency was not available, it was assumed that CD31 positive cell make up the majority of the CD8$^+$ circulating T cells, and was set to $R = 0.98$ for all donors.
- Total cell number C. The total cell number was not available for this study, but was determined by quadratic regression from published data [Fagnoni et al. (2001)]. Data are found in `S Table 1`, table `naive_CD8_number`.

A overview of the data used for fitting CD8$^+$ T cell parameters is shown in S Fig 2A.

The linear problem for the CD8$^+$ T cells is

$$\begin{bmatrix} A & 0 & 0 & 0 \\ -TC/T_0 & 0 & 0 & 1 \\ -CR & -2CR & CR & 1 \\ -C(1-R) & 2CR & 0 & 0 \end{bmatrix} \begin{bmatrix} \gamma \\ \rho \\ \rho\delta \\ F \end{bmatrix} = \begin{bmatrix} 1 \\ 0 \\ 0 \\ 0 \end{bmatrix}. \tag{26}$$

A unique solution could be found, excepted in four donors where the average cell age was $A = 0$. Then, γ was set arbitrarily to 100.0 per year; and the parameters were considered outliers for the linear regressions in Fig 4B.

Robustness analysis

Robustness to noise to the conclusion “There is no long-lived naive T cell pool”

We tested the conclusion that there is no long-lived cell pool in the $CD4^+$ and $CD8^+$ naive T cell populations. The conclusion rested on the comparison of two models for T cell turnover. In the first one, the turnover was assumed to be the same for all cells, i.e. all cell are equally likely to be replaced (ASE, model A). In the second one, a subset (of unknown size) of the cells are never replaced, leading to a long-lived pool (ASE, model 2POPA). Required data are the dates of birth of the donors, the date of collection of the samples, and the 14C levels of the samples (all available in `S1 Data`, table `radiocarbon_samples`).

To test the robustness of the conclusion that there is no long-lived naive T cell pool, we perturbed the samples 14C levels by random factors proportional to the measurement error (errors given in `S1 Data`, table `radiocarbon_samples`, column `Delta14C error (2*std)`). For example, a noise level of 0.5 means that the 14C levels have been perturbed by a random Gaussian number with standard deviation equal to `0.5*Delta14C_ERROR`.

Simulations were repeated 100 times for each cell type/noise level combination (100 * 4 cell types * 4 noise levels = 1600 runs).

Table 3: Robustness measurements for testing the existence of non-dividing populations of naive T cells.

TYPE/NOISE	0.0	0.5	1.0	2.0	5.0
$CD31^+$	1.0	0.99	0.87	0.79	0.78
$CD31^-$	1.0	0.96	0.90	0.82	0.85
$CD8^+$	1.0	0.99	0.84	0.72	0.76
$CD4^+$	1.0	0.98	0.87	0.73	0.69

Table 3 reports, for each cell type/noise level, the fraction of times model A was selected. For noise levels below 1.0, there was no long-lived cell pool in at least 84% of the simulations. Even at 5.0 times the normal error, the conclusion held most of the time (more than 69% of the time).

Robustness to noise to the conclusion “There is a linear correlation in T cell age vs donor age”

We tested the conclusion that there is a linear correlation between average cell age and donor age (`S Table 3`, table `average cell ages`). To test the robustness of the conclusion, we perturbed the sample 14C levels by random

factors proportional to the measurement error (errors given in `S1 Data`, table `radiocarbon_samples`, column `Delta14C error (2*std)`).

Simulations were repeated 100 times for each cell type/noise level combination (100 * 4 cell types * 3 noise levels = 1200 runs).

Table 4: Robustness measurements concerning the impact of 14C measurement noise to the conclusions that a linear correlation exists between donor age and naive T cell age.

TYPE/NOISE	0.0	0.5	1.0	2.0	5.0
$CD31^{+}$	1.00	0.97	0.54	0.33	0.16
$CD31^{-}$	1.00	0.82	0.52	0.12	0.08
$CD8^{+}$	1.00	1.00	0.86	0.44	0.21
$CD4^{+}$	1.00	1.00	0.88	0.45	0.14

Table 4 reports, for each cell type/noise levels, the fraction of times a significant (p-value<0.05) correlation was obtained. For noise levels below 0.5, there was a linear correlation in at least 82% of the simulations. The fraction of correlations dropped sharply for higher noise levels. Yet, at noise level of 5.0, the fraction of correlation was still above the significance cut-off p-value=0.05, showing that there was still detectable signal the data.

Robustness to donor permutation to the conclusion "There is a linear correlation in T cell age vs donor age"

To exclude a possible effect of the date of birth, we also ran simulation with permuted dates of birth. After permutation, there should be no correlation left, so any residual correlation should be regarded as a bias in the cell age estimation method.

Permutations were repeated 100 times for each cell type (100 * 4 cell types = 400 runs).

Table 5: Robustness measurements concerning the impact of donor birth date to the conclusions that a linear correlation exists between donor age and naive T cell age.

TYPE	Fraction with p-value < 0.05
$CD31^{+}$	0.06
$CD31^{-}$	0.06
$CD8^{+}$	0.06
$CD4^{+}$	0.08

Table 5 reports, for each cell type, the fractions of simulations with p-values below the 0.05 threshold. The 95% percent confidence interval around 0.05 over 100 trials is $p \in [0.035 to 0.15]$, so the frequencies reported in Table 5 did not differ from chance. We conclude that the linear correlation seen in the data is real and not due to a bias in method.

Robustness to noise to the conclusion "Parameter identification indicates that $CD31^-$ T cell do not proliferate"

We concluded, based on model selection, that the $CD31^-$ T cells do not proliferate. To test the robustness of this conclusion, we perturbed the average cell ages, TREC contents, cell counts, and $CD31^+$ cell fractions by random factors.

Simulations were repeated 100 times for each model/noise level combination (100 * 5 models * 7 noise levels = 3500 runs).

Table 6: Robustness measurements relative to model selection for Figure 3B concerning best-fit determination relative to $CD31^-$ proliferation assumptions.

TYPE/NOISE	0.0	0.05	0.1	0.2	0.3	0.4	0.5	1.0
Model I	0.00	0.00	0.00	0.00	0.00	0.00	0.00	0.00
Model II	1.00	1.00	1.00	0.98	0.86	0.57	0.24	0.03
Model III	0.00	0.00	0.00	0.00	0.00	0.00	0.00	0.00
Model IV	0.00	0.00	0.00	0.02	0.14	0.43	0.76	0.97
Model V	0.00	0.00	0.00	0.00	0.00	0.00	0.00	0.00

Table 6 reports, for each model/noise level, the fraction of times the model was accepted. For noise levels below or equal to 0.3, model II was accepted most of the times. For noise levels equal or above, model IV was accepted most of the time. Other models (I, III, V) were never selected.

We conclude that a model in which $CD31^-$ cells do not proliferate can explain the data adequately. These results also indicate that if data are noisy enough the conclusion that $CD31^-$ do no proliferate does not hold anymore.
However, uncertainties on $CD31^+$ fractions and other marker specific data points could blur the line between naive $CD4^+/CD31^+$ and $CD4^+/CD31^-$ cells, leading to the conclusion that $CD31^-$ cells do in fact proliferate.

References

I Bains, R Antia, R Callard, AJ Yates (2009) Quantifying the development of the peripheral naive CD4+ T-cell pool in humans. Blood 113:5480-5487

DC Douek, MR Betts, BJ Hill, SJ Little, R Lempicki, JA Metcalf, J Casazza, C Yoder, JW Adelsberger, RA Stevens, ... (2001) Evidence for increased T cell turnover and decreased thymic output in HIV infection. The Journal of Immunology 167:6663-6668

KP Burnham, DR Anderson (2002) Model selection and multimodel inference: a practical information-theoretic approach. Springer-Verlag New York.

I den Braber, T Mugwagwa, N Vrisekoop, L Westera, R Mögling, AB de Boer, N Willems, EH Schrijver, G Spierenburg, K Gaiser, ... (2012) Maintenance of peripheral naive T cells is sustained by thymus output in mice but not humans. Immunity 36:288-297

FF Fagnoni, R Vescovini, G Passeri, G Bologna, M Pedrazzoni, G Lavagetto, A Casti, C Franceschi, M Passeri, P Sansoni (2000) Shortage of circulating naive CD8+ T cells provides new insights on immunodeficiency in aging. Blood 95:2860-2868

RD Kilpatrick, T Rickabaugh, LE Hultin, P Hultin, MA Hausner, R Detels, J Phair, BD Jamieson (2008) Homeostasis of the naive CD4+ T cell compartment during aging. The Journal of Immunology 180:1499-1507
